# Re-evaluating the impact and cost-effectiveness of pneumococcal conjugate vaccine introduction in 112 low-income and middle-income countries in children younger than 5 years: a modelling study

**DOI:** 10.1016/S2214-109X(24)00232-8

**Published:** 2024-08-14

**Authors:** Cynthia Chen, Gregory Ang, Katika Akksilp, Jemima Koh, J Anthony G Scott, Andrew Clark, Mark Jit

**Affiliations:** a Saw Swee Hock School of Public Health, National University of Singapore and National University Health System, Singapore; b Yong Loo Lin School of Medicine, National University of Singapore, Singapore; c Schaeffer Center for Health Policy and Economics, University of Southern California, CA, USA; d Department of Health Service Research, Changi General Hospital, Singapore; e Department of Infectious Disease Epidemiology, Faculty of Epidemiology and Population Health, London School of Hygiene & Tropical Medicine, London, UK; f Department of Health Services Research and Policy, Faculty of Public Health and Policy, London School of Hygiene & Tropical Medicine, London, UK

## Abstract

**Background:**

*Streptococcus pneumoniae* has been estimated to cause 9·18 million cases of pneumococcal pneumonia, meningitis, and invasive non-pneumonia non-meningitis disease and 318 000 deaths among children younger than 5 years in 2015. We estimated the potential impact and cost-effectiveness of pneumococcal conjugate vaccine (PCV) introduction.

**Methods:**

We updated our existing pseudodynamic model to estimate the impact of 13-valent PCV (PCV13) in 112 low-income and middle-income countries by adapting our previously published pseudodynamic model with new country-specific evidence on vaccine coverage, burden, and post-introduction vaccine impact from WHO–UNICEF estimates of national immunisation coverage and a global burden study. Deaths, disability-adjusted life-years (DALYs), and cases averted were estimated for children younger than 5 years born between 2000 and 2030. We used specific PCV coverage in each country and a hypothetical scenario in which coverage increased to diphtheria–tetanus–pertussis (DTP) levels. We conducted probabilistic uncertainty analyses.

**Findings:**

Using specific vaccine coverage in countries, we estimated that PCV13 could prevent 697 000 (95% credibility interval 359 000–1 040 000) deaths, 46*·*0 (24*·*0–68*·*9) million DALYs, and 131 (89*·*0–172) million cases in 112 countries between 2000 and 2030. PCV was estimated to prevent 5*·*3% of pneumococcal deaths in children younger than 5 years during 2000–30. The incremental cost of vaccination would be I$851 (510–1530) per DALY averted. If PCV coverage were increased to DTP coverage in 2020, PCV13 could prevent an additional 146 000 (75 500–219 000) deaths.

**Interpretation:**

The inclusion of real-world evidence from lower-income settings revealed that delays in PCV roll-out globally and low PCV coverage have cost many lives. Countries with delays in vaccine introduction or low vaccine coverage have experienced many PCV-preventable deaths. These findings underscore the importance of rapidly scaling up PCV to achieve high coverage and maximise vaccine impact.

**Funding:**

Bill & Melinda Gates Foundation and Gavi, the Vaccine Alliance.

## Introduction

*Streptococcus pneumoniae* is a species of bacteria that causes various diseases, including pneumonia, meningitis, acute otitis media, and non-pneumonia, non-meningitis invasive disease (NPNM), which is pneumococcal infection in normally sterile body fluid, presenting without pneumonia or meningitis. Pneumococcal infections are clinically categorised as invasive pneumococcal disease (IPD), where infection occurs in a normally sterile site, or non-invasive pneumococcal disease (nIPD), such as otitis media or pneumonia without bacteraemia.^1^ These diseases can impose large burdens on health-care systems and lead to deaths when not treated.

Global modelling has estimated that there were 9*·*18 million cases and 318 000 deaths due to pneumococcal disease among children younger than 5 years in 2015.^2^ In 2016, pneumococcus remained the main contributor to global lower respiratory tract infection.^3^ However, the disease burden has decreased following the introduction of the pneumococcal conjugate vaccine (PCV) in routine infant immunisation, as recommended by WHO in 2007.^4^ The introduction of PCVs has effectively reduced the burden of diseases, especially in children younger than 5 years. Previous modelling studies have suggested that PCVs could avert at least 69 million deaths in low-income and middle-income countries (LMICs)^5^, ^6^ between 2000 and 2030, based on projected vaccine uptake to high levels of coverage. 143 countries have introduced PCVs into their national immunisation programmes, including 60 of 73 countries eligible for Gavi, the Vaccine Alliance support.^7^ The two main PCVs currently used in infant immunisation programmes are ten-valent PCV (PCV10, 26 countries) and 13-valent PCV (PCV13, 111 countries), with others using a mix of PCV10 and PCV13 (6 countries). Countries using PCV10 are mostly using Synflorix (GlaxoSmithKline, London, UK). The remaining disease burden is affected by delays in PCV introduction in LMICs and low vaccine coverage in countries with existing PCV programmes.

**Research in context**

**Evidence before this study**

Pneumococcal disease is a leading cause of childhood mortality worldwide. Although pneumococcal conjugate vaccines (PCVs) have been available for more than two decades, a quarter of low-income and middle-income countries globally have not introduced PCVs to their childhood immunisation programmes. We searched PubMed for publications between database inception and July 28, 2022, using terms associated with “pneumococcal conjugate vaccine”, “child*”, “modelling*”, “global”, and “estimate*” without restrictions. We also supplemented this search with studies already known to the authors. We found three global modelling studies. Two studies did not account for herd effects or include an economic evaluation; one study used vital registration data for countries where data were available; and one study focused solely on mortality in children younger than 5 years when estimating the global disease burden. The third study was our 2019 study and it incorporated herd effects. 13-valent PCV vaccination was estimated in our 2019 study to prevent 34% of global deaths (0·4 million child deaths) and 12% of cases (54·6 million cases) annually in 180 countries. Additionally, the previous analysis reported that global vaccine costs of I$15·5 billion could be partially balanced by health-care savings of $3·19 billion. Systematic reviews and meta-analyses also support that ten-valent and 13-valent PCVs are cost-effective in studies from various nations, with the most influential parameters being vaccine efficacy and coverage in the countries.

**Added value of this study**

Post-PCV introduction studies in low-income and middle-income countries have found that, although the prevalence of vaccine-type carriage has reduced following PCV introduction, the non-vaccine-type carriage has increased. Our study estimated the impact of PCV introduction by considering both the direct and indirect (herd effects and serotype replacement) effects of vaccination. We combined our previous pseudo-dynamic model of herd immunity and serotype replacement with new data to evaluate the impact of PCV introduction in 112 low-income and middle-income countries, including evidence gathered post-introduction and meta-analyses to quantify the disease dynamics of pneumococcal diseases better. We found that PCV introduction, with an incremental cost of I$851 per disability-adjusted life year averted, has the potential to reduce the disease burden and that there is room for outcomes averted by vaccination to double compared with the situation in 2022.

**Implications of all the available evidence**

Our study provides updated evidence that PCVs could potentially reduce the burden of pneumococcal diseases, although the greatest impact will only be reached with rapid scale-up to high vaccine coverage. Delays in vaccine introduction and lower PCV coverage rates in low-income and middle-income countries have cost the lives of many children younger than 5 years. Our findings highlight the importance of improving PCV coverage and financing Gavi, the Vaccine Alliance-funded programmes for countries that have yet to introduce PCV to their childhood immunisation.

Mathematical models can project the health and economic benefits of PCV roll-out globally. However, most multi-country models of PCV impact have only assessed the direct effect of vaccination.^8^, ^9^ The most widely used model is the static UNIVAC model, a decision-support tool for evaluating the cost-effectiveness of vaccine introduction.^10^ The biggest limitation of UNIVAC and other static models is that they do not account for the positive and negative indirect ecological impacts of PCVs, such as herd effects and serotype replacement.^11^

In 2019, we published a global effect and cost-effectiveness analysis of PCV incorporating indirect ecological effects.^8^ In this analysis, we used a pseudodynamic approximation that assumed the complete elimination of vaccine-type pneumococcal carriage in children younger than 5 years and complete replacement by non-vaccine-type carriage, irrespective of the vaccine coverage in each country.^12^ These assumptions were found to be realistic in high-income settings where vaccine coverage levels are high. Under these assumptions, we predicted a 34% reduction in pneumococcal deaths in children younger than 5 years in 180 countries from 2000 to 2030.^8^ However, until recently, there have been few studies after vaccine introduction in LMICs. For example, Mongolia's district-level coverage and carriage data suggested that, as vaccine coverages increase from 0% to 100%, carriage due to serotypes in PCV13 will decrease from 29*·*1% to 13*·*1%.^13^ Similar data from Laos found that increasing PCV13 coverage from 0% to 60% could lead to a reduction in vaccine-type carriage from 20*·*0% to 12*·*8%.^14^ In two different settings in Nigeria, increasing PCV10 coverage in children younger than 5 years from 7% to 84% in a rural area could lead to a decrease in vaccine-type carriage from 21% to 12%.^15^

In this Article, we update our previous estimates of the impact of PCV introduction in 112 LMICs, accounting for serotype replacement and herd protection, but adjusting assumptions based on emerging evidence of the real-world impact of PCV vaccine coverage.

## Methods

### Model design

We updated our existing pseudodynamic model^8^ with countries’ coverage, where both ecological and decision-tree models were used to generate new estimates of the impact of PCVs on cases and deaths (and associated disability-adjusted life years [DALYs]) in children younger than 5 years in 112 countries over 30 years (2000–30). The estimates of cases and deaths in the no vaccination scenario were multiplied by the predicted incidence risk ratio (IRR) from an updated pseudodynamic algorithm, accounting for four new characteristics. We then compared estimates from no vaccination and vaccination scenarios.

Disease pathways for a birth cohort with and without vaccination are described in the appendix (p 3). Under the no vaccination scenario, we estimated disease burden (ie, cases, deaths, and DALYs) for each birth cohort (from birth to age younger than 5 years) by multiplying country-specific disease incidence and mortality^2^ by the size of each birth cohort. We modelled severe and non-severe pneumonia and NPNM, with deaths only from severe diseases and meningitis. The risk of disabling sequelae from pneumococcal meningitis was obtained from a review.^16^ Regional acute otitis media incidence was obtained from a global systematic review,^17^ where 20% of the incidence was assumed to be attributed to *S pneumoniae*.^18^ Disability weights for the exact form of sequelae were obtained from the Global Burden of Disease study,^19^ wherever possible. We used the disability weights for moderate lower respiratory infection as a proxy for non-severe pneumonia and non-severe NPNM, and the disability weights for severe lower respiratory infection as a proxy for severe pneumonia. Severe NPNM was assumed to have the same weight as meningitis. Input values and ranges for each country for these parameters are available in the appendix (pp 10–15).

Our previous model^8^ included four categories of disease outcomes attributable to *S pneumoniae:* pneumonia, meningitis, NPNM, and acute otitis media. The impact of PCV introduction on IPD was estimated using a pseudodynamic model, which projected the long-term predicted IRR following PCV introduction using a single equation.^12^ The predicted IRRs from the pseudodynamic model^12^ were specific to six UN regions (Africa, Asia, Europe, Latin America and the Caribbean, North America, and Oceania) and we assumed that countries in the same region would have the same IRRs. Countries categorised in each UN region are available in the appendix (pp 7–10).

Our updated model incorporates four additional characteristics (appendix pp 4–6): country-specific input parameters, including actual PCV coverage; time taken to the near elimination of vaccine-type IPD; vaccine coverage required to reach full vaccine impact; and differentiated PCV impact on nIPD. We incorporated recent real-world evidence on the extent of herd immunity and serotype replacement in settings with moderate vaccine coverage and the time it takes to reach a new post-vaccine introduction equilibrium in vaccine-type carriage.^10^, ^20^ In addition, as evidence of PCV on nIPD was unclear, we assembled an expert panel, selected based on personal knowledge and networks of the authors, to provide input on the parameters and assumptions to be used in the model, particularly on the impact of PCVs on nIPD. Our analysis focuses on a vaccine that prevents the serotypes present in PCV13, which is the most common vaccine used globally.

Ethical approval for this study was obtained from the Institutional Review Board of the National University of Singapore (NUS-IRB-2022-582).

### Country-specific input parameters

All countries’ demography inputs (total population, life expectancy, and mortality by age) were based on the UN World Population Prospects 2019.^21^ Country-specific disease incidence and mortality rates for pneumonia, meningitis, and NPNM were obtained from a global burden study (appendix pp 11–14).^2^ Real-world, national-level PCV coverage estimates up to 2019 were obtained from WHO–UNICEF estimates of national immunisation coverage.^22^ In this model, we assumed a three-dose schedule for PCV13 and that the dose-specific coverage estimates have the same drop-out rates as the three-dose diphtheria–tetanus–pertussis (DTP; DTP3) vaccine, as reported by WHO–UNICEF (where two-dose DTP coverage was assumed to be the mean coverage of one-dose DTP and DTP3).^23^

We considered two scenarios to project the coverage from 2020 to 2030. First, we adopted a conservative approach using 2019 PCV coverage in each country for future years (appendix pp 3–4). In the second scenario, we considered a best-case approach, where vaccine coverage in children younger than 1 year from 2020 to 2030 for each country would increase to their respective DTP coverage levels in 2019. The coverage in children younger than 5 years of a particular year would be the weighted (by cohort size) coverage in children younger than 1 year of that year and the preceding 4 years.

We used our existing pseudodynamic model to project vaccine impact on IPD, and we tracked subsequent disease and health-care burden consequences of the IPD projections from our pseudodynamic model. However, we updated this model in consultation with pneumococcal disease experts to account for recent data post-introduction suggesting that the time taken for vaccination to nearly eliminate vaccine-type IPD is longer than 2 years, that high vaccine coverage is needed to reach maximum vaccine impact, and that PCVs have a smaller effect on nIPD (pneumonia and acute otitis media) compared with IPD (appendix pp 4–7).^20^ All unit prices were converted to 2015 international dollars (I$).

### Statistical analysis

In a probabilistic sensitivity analysis sources of uncertainty in the model were explored by randomly drawing 1000 samples from country-specific distributions for parameters that were parameterised by their respective low, mid, and high values (appendix pp 15–16). The predicted IRR from the pseudo-dynamic model was obtained by bootstrap sampling.^12^ Mean and 95% CI of outcomes outputs from using each of the 1000 iterations were reported.

We also performed three additional comparisons. First, we compared the previous 2019 model^8^ with real-world PCV coverage and the year of vaccination introduction. Second, we compared the current updated model and DTP vaccine coverage with UNIVAC,^10^ a commonly used model (appendix pp 20–27).

We also performed an economic evaluation. Using the decision-tree model (appendix p 3), we compared three vaccination strategies with the no vaccination scenario: PCV coverage, DTP coverage, and a full protection scenario from a health-system perspective. We evaluated the cost-effectiveness by comparing the no vaccination scenario with the PCV coverage scenario and the full protection scenario. The incremental cost-effectiveness ratio (ICER) of PCV introduction was defined as the discounted incremental cost of PCV introduction from 2000 to 2030 years divided by the discounted incremental DALYs averted by vaccination over the same period. We applied similar guidelines as those provided by Lomas and colleagues,^24^ adopting a constant discount rate of 3% per annum for all countries, which was applied to all costs and effects.^25^, ^26^, ^27^, ^28^, ^29^ We used I$500 as a lower bound threshold based on willingness to pay in respondents to reduce the risk of meningitis^30^ and we increased the threshold to $5000 to facilitate comparisons with our previous findings.^8^ More recent estimates using econometric methods to estimate health opportunity costs suggest cost-effectiveness thresholds of less than one gross domestic product per capita. Thus, we used thresholds estimated in each country from a 2023 study.^31^ We compared the countries’ ICER to their respective cost-effectiveness thresholds per life-year, estimated on the basis of per-capita health expenditures and life expectancy.^31^

We did one-way scenario and probabilistic sensitivity analyses to test the robustness of our model results to changes in key parameters over plausible ranges. For one-way analyses, we assessed the effect of varying disease incidence and case-fatality rates between the lower and upper 95% credible intervals (95% CrIs). We also varied the vaccine price and health-care cost parameters by 20%. Discounts were also varied between 0% and 6%. All analyses were done in R (3.6.3).

### Role of the funding source

The funders were given the opportunity to review this paper before publication, but the final decision on the content of the publication was taken by the authors. The funders of the study had no role in study design, data collection, data analysis, data interpretation, or writing of the report.

## Results

Countries were categorised into the African (48 countries), Asian (31 countries), European (eight countries), Latin American and the Caribbean (15 countries), and Oceania (ten countries) regions. Based on WHO–UNICEF data, countries had different PCV introduction years. As of 2019, 27*·*7% (31 countries) of the countries had yet to introduce PCVs in their routine infant vaccination schedule. Information on PCV introduction by country is detailed in the appendix (pp 7–10).

**Figure 1:**
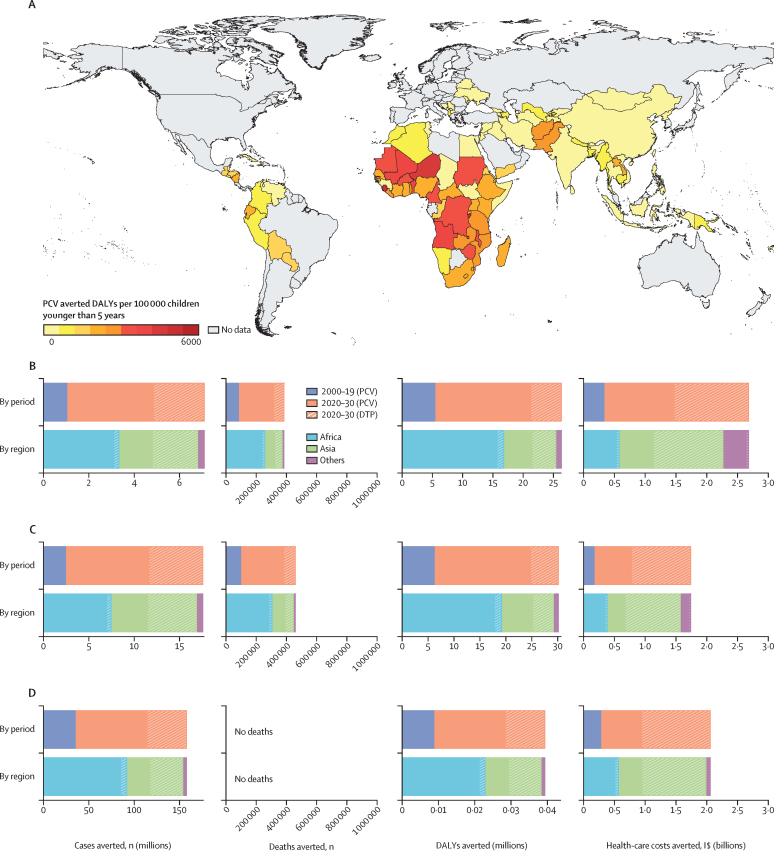
Cases, deaths, and DALYs averted compared with no vaccination scenario, by countries, regions, and time period (undiscounted) (A) DALYs averted per 100 000 children younger than 5 years using real-world vaccine coverage from years 2000 to 2030 by country. Cases averted for invasive pneumococcal diseases (B), non-invasive pneumococcal diseases (C), and acute otitis media (D). x axes reflect the cases, deaths, DALYs averted, and health-care costs saved with PCV roll-out for each disease. The outcomes are stratified by time periods (2000 to 2019 and 2020 to 2030) and by regions (Africa, Asia, and others [Europe, Latin America and the Caribbean, and Oceania]). Health-care costs arise from hospitalisation and outpatient visits. For by period data, the dark blue solid areas represent the outcomes averted at PCV coverage levels from 2000 to 2019 and the orange solid areas from 2020 to 2030. The orange diagonal stripes represent the additional outcomes averted when vaccine coverage levels were increased to DTP levels from 2020 to 2030. For by region data, the light blue, green, and purple solid areas represent the outcomes averted at PCV coverage levels from 2000 to 2030 in Africa, Asia, and others (Europe, Latin America and the Caribbean, and Oceania) respectively. The light blue, green, and purple diagonal stripes represent the additional outcomes averted when vaccine coverage levels were increased to DTP levels from 2020 to 2030 in Africa, Asia, and others (Europe, Latin America and the Caribbean, and Oceania). DALY=disability-adjusted life year. DTP=diphtheria–tetanus–pertussis. I$=international dollars. PCV=pneumococcal conjugate vaccine.

Using countries’ vaccine coverage data, the African region averted 72*·*9% of all cases, 75*·*1% of all deaths, and 73*·*0% of all DALYs (figure 1A–C). This was followed by Asia with 23*·*9% of all cases, 21*·*6% of all deaths, and 23*·*3% of all DALYs averted (figure 1C). PCV introduction in the African region had the largest effect compared with other regions, averting a total of 95*·*2 million cases (95% CrI 64*·*0 million to 126 million), 524 000 (201 000–852 000) deaths, and 33*·*5 (12*·*9–54*·*7) million DALYs. We estimated a total of 92*·*1 (63*·*1–121) million cases, 517 000 (267 000–772 000) deaths, and 34*·*4 (18*·*2–51*·*4) million DALYs to be averted globally from 2020 to 2030 due to the impact of PCVs (table 1). These estimations were higher than those of the predictions averted in the 2000–19 period (figure 1B). Acute otitis media had the largest number of cases averted, at 114 (73*·*7–155) million cases globally, whereas nIPD pneumonia had the largest number of deaths averted at 382 000 (208 000–563 000) and 24*·*7 (13*·*6–36*·*3) million DALYs averted (figure 1C).

**Table 1:** Cases, deaths, and DALYs averted and health-care costs saved by regions and time periods (undiscounted)

		**No vaccination minus PCV introduction (2000–30)**				**No vaccination minus PCV introduction (2020–30)**				**PCV introduction minus PCV introduction at DTP coverage (2020–30)**			
		Cases, n (millions)	Deaths, n	DALYs (millions)	Health-care costs, I$ (billions)	Cases, n (millions)	Deaths, n	DALYs (millions)	Health-care costs, I$ (billion)	Cases, n (millions)	Deaths, n	DALYs (millions)	Health-care costs, I$ (billions)
Global		131 (89·0–172)	697 000 (359 000–1 040 000)	46·0 (24·0–68·9)	3·19 (2·19–4·30)	92·1 (63·1–121)	517 000 (267 000–772 000)	34·4 (18·2–51·4)	2·41 (1·62–3·26)	51·7 (34·7–68·2)	146 000 (75 500–219 000)	10·5 (5·38–16·1)	3·29 (1·92–4·62)
	IPD	4·85 (2·70–7·17)	315 000 (150 000–509 000)	21·2 (10·3–34·1)	1·47 (0·812–2·24)	3·82 (2·12–5·63)	233 000 (113 000–373 000)	15·8 (7·68–25·3)	1·14 (0·616–1·75)	2·24 (1·05–3·49)	68 400 (32 600–110 000)	5·09 (2·43–8·34)	1·21 (0·523–2·04)
	nIPD pneumonia	11·6 (6·76–16·4)	382 000 (208 000–563 000)	24·7 (13·6–36·3)	0·775 (0·505–1·05)	9·19 (5·36–13·0)	284 000 (155 000–419 000)	18·5 (10·2–27·0)	0·605 (0·386–0·827)	5·96 (2·70–9·13)	77 600 (42 100–113 000)	5·41 (2·85–7·96)	0·963 (0·418–1·50)
Acute otitis media		114 (73·7–155)	0	0·0285 (0·0184–0·0386)	0·944 (0·606–1·28)	79·1 (51·0–107)	0	0·0197 (0·0127–0·0268)	0·665 (0·426–0·902)	43·5 (27·5–59·2)	0	0·0108 (0·00687–0·0148)	1·11 (0·701–1·52)
Africa		95·2 (64–126)	524 000 (201 000–852 000)	33·5 (12·9–54·7)	1·39 (0·789–1·98)	65·7 (44·3–87·0)	382 000 (146 000–622 000)	24·7 (9·48–40·3)	1·01 (0·564–1·46)	7·24 (4·89–9·57)	38 400 (14 800–62 700)	2·43 (0·939–3·98)	0·138 (0·0808–0·194)
	IPD	3·11 (1·18–5·11)	240 000 (88 600–411 000)	15·7 (5·78–27·1)	0·535 (0·197–0·915)	2·38 (0·908–3·92)	174 000 (64 300–298 000)	11·5 (4·22–19·8)	0·408 (0·150–0·698)	0·218 (0·0837–0·359)	17 100 (6330–29 700)	1·10 (0·407–1·92)	0·0430 (0·0159–0·0735)
	nIPD pneumonia	6·96 (2·81–11·1)	284 000 (111 000–457 000)	17·8 (6·96–28·7)	0·347 (0·140–0·554)	5·34 (2·16–8·53)	208 000 (81 200–336 000)	13·2 (5·14–21·2)	0·259 (0·105–0·414)	0·502 (0·202–0·803)	21 200 (8290–34 200)	1·33 (0·52–2·15)	0·0382 (0·0154–0·0609)
	Acute otitis media	85·1 (55·3–115)	0	0·0212 (0·0138–0·0288)	0·509 (0·330–0·689)	58 (37·6–78·6)	0	0·0145 (0·00938–0·0196)	0·343 (0·222–0·465)	6·51 (4·26–8·79)	0	0·00162 (0·00106–0·00219)	0·0566 (0·0363–0·0763)
Asia		31·3 (20·9–41·3)	151 000 (58 900–247 000)	10·7 (4·19–17·6)	1·23 (0·660–1·83)	23·8 (15·9–31·2)	120 000 (46 600–196 000)	8·54 (3·34–14·1)	1·03 (0·532–1·54)	43·6 (29·0–57·6)	106 000 (41 000–175 000)	7·97 (3·13–13·2)	3·06 (1·70–4·38)
	IPD	1·48 (0·577–2·41)	64 700 (25 000–113 000)	4·69 (1·82–8·24)	0·558 (0·213–0·992)	1·25 (0·488–2·04)	51 700 (20 000–90 700)	3·78 (1·46–6·64)	0·478 (0·183–0·850)	1·99 (0·771–3·24)	50 500 (19 800–88 300)	3·93 (1·54–6·90)	1·13 (0·447–1·94)
	nIPD pneumonia	4·03 (1·59–6·47)	86 100 (33 100–139 000)	6·00 (2·31–9·66)	0·289 (0·114–0·464)	3·42 (1·34–5·49)	67 900 (26 100–109 000)	4·76 (1·83–7·67)	0·255 (0·100–0·409)	5·38 (2·11–8·62)	55 500 (21 300–89 200)	4·02 (1·54–6·46)	0·899 (0·351–1·44)
	Acute otitis media	25·8 (16·3–35·1)	0	0·00642 (0·00407–0·00875)	0·383 (0·243–0·522)	19·1 (12·0–26·0)	0	0·00476 (0·00300–0·00649)	0·292 (0·185–0·397)	36·2 (22·8–49·4)	0	0·00903 (0·00568–0·0123)	1·04 (0·652–1·41)
Europe		0·0647 (0·0431–0·0854)	294 (197–415)	0·0232 (0·0152–0·0334)	0·00547 (0·00318–0·00886)	0·0396 (0·0267–0·0522)	199 (133–281)	0·0157 (0·0103–0·0227)	0·00382 (0·00216–0·00628)	0·310 (0·211–0·408)	164 (108–244)	0·0133 (0·00838–0·0203)	0·0220 (0·0169–0·0281)
	IPD	0·00216 (0·00141–0·00323)	129 (58–226)	0·0105 (0·00446–0·0188)	0·00346 (0·00131–0·00667)	0·00162 (0·00107–0·00240)	87 (39–153)	0·00711 (0·00304–0·0128)	0·00252 (0·000962–0·00483)	0·00729 (0·00550–0·00934)	60 (26–126)	0·00548 (0·00230–0·0115)	0·00813 (0·00490–0·0125)
	nIPD pneumonia	0·00454 (0·00365–0·00543)	166 (133–198)	0·0127 (0·0102–0·0153)	0·000970 (0·000781–0·00116)	0·00344 (0·00277–0·00412)	112 (90–134)	0·00861 (0·00689–0·0103)	0·000710 (0·000573–0·000851)	0·0205 (0·0165–0·0247)	104 (79–126)	0·00774 (0·00590–0·00935)	0·00788 (0·00634–0·00947)
	Acute otitis media	0·0580 (0·0368–0·0787)	0	0·0000145 (0·00000917–0·0000196)	0·00104 (0·000660–0·00141)	0·0346 (0·0219–0·0470)	0	0·00000862 (0·00000545–0·0000117)	0·000590 (0·000373–0·000801)	0·282 (0·185–0·381)	0	0·0000702 (0·0000460–0·0000949)	0·00595 (0·00386–0·00805)
Latin America and the Caribbean		3·64 (2·63–4·63)	20 700 (14 500–27 300)	1·61 (1·12–2·15)	0·547 (0·373–0·752)	2·24 (1·64–2·83)	13 700 (9590–18 000)	1·06 (0·738–1·42)	0·356 (0·240–0·491)	0·491 (0·345–0·634)	1340 (867–1860)	0·101 (0·0641–0·142)	0·0624 (0·0450–0·0823)
	IPD	0·249 (0·185–0·322)	9770 (5530–14 800)	0·787 (0·438–1·20)	0·362 (0·207–0·546)	0·170 (0·126–0·220)	6400 (3620–9680)	0·516 (0·287–0·788)	0·240 (0·136–0·362)	0·0196 (0·0147–0·0253)	697 (340–1120)	0·0539 (0·0257–0·0883)	0·0305 (0·0167–0·0476)
	nIPD pneumonia	0·594 (0·486–0·7)	10 900 (8890–12 800)	0·822 (0·668–0·965)	0·135 (0·110–0·159)	0·405 (0·331–0·477)	7260 (5910–8520)	0·546 (0·444–0·640)	0·0877 (0·0717–0·103)	0·0508 (0·0415–0·0599)	646 (514–763)	0·0469 (0·0374–0·0555)	0·0180 (0·0147–0·0212)
	Acute otitis media	2·79 (1·79–3·77)	0	0·000696 (0·000447–0·000940)	0·0497 (0·0318–0·0671)	1·66 (1·07–2·24)	0	0·000414 (0·000266–0·000560)	0·0283 (0·0181–0·0383)	0·421 (0·275–0·564)	0	0·000105 (0·0000686–0·000141)	0·0139 (0·00904–0·0186)
Oceania		0·472 (0·304–0·627)	1520 (502–2560)	0·105 (0·0345–0·178)	0·0154 (0·00608–0·0275)	0·365 (0·234–0·484)	1320 (437–2230)	0·0915 (0·0301–0·155)	0·0127 (0·00499–0·0228)	0·0357 (0·0229–0·0477)	37 (12–63)	0·00279 (0·000950–0·00487)	0·000860 (0·000359–0·00147)
	IPD	0·0120 (0·00401–0·0205)	609 (206–1130)	0·0434 (0·0148–0·0818)	0·0112 (0·00369–0·0219)	0·0105 (0·00350–0·0179)	527 (179–980)	0·0376 (0·0127–0·0709)	0·00928 (0·00301–0·0182)	0·000772 (0·000257–0·00130)	17 (6–33)	0·00138 (0·000450–0·00269)	0·000523 (0·000174–0·00100)
	nIPD pneumonia	0·0287 (0·00966–0·0463)	913 (307–1460)	0·0614 (0·0207–0·0982)	0·00270 (0·000909–0·00435)	0·0253 (0·00851–0·0407)	798 (269–1280)	0·0538 (0·0181–0·0861)	0·00235 (0·000791–0·00378)	0·00201 (0·000677–0·00324)	20 (7–31)	0·00140 (0·000472–0·00224)	0·000235 (0·0000791–0·000378)
Acute otitis media		0·432 (0·271–0·585)	0	0·000108 (0·0000675–0·000146)	0·00147 (0·000919–0·00199)	0·329 (0·206–0·446)	0	0·0000821 (0·0000514–0·000111)	0·00109 (0·000680–0·00148)	0·0330 (0·0206–0·0447)	0	0·00000822 (0·00000513–0·0000112)	0·000102 (0·0000639–0·000138)

Data are in mean (95% credible interval). Health-care costs arise from hospitalisation and outpatient visits. Inclusive of only 112 low-income and middle-income countries. DALY=disability-adjusted life year. DTP=diphtheria–tetanus–pertussis. IPD=invasive pneumococcal disease (pneumococcal meningitis, pneumococcal non-pneumonia, non-meningitis, and invasive pneumococcal pneumonia). I$=international $. nIPD=non-invasive pneumococcal disease. PCV=pneumococcal conjugate vaccine.

There were 31 countries with no PCV vaccination programmes in 2019, including countries with high rates of PCV-preventable deaths, such as Egypt and South Sudan. We estimated that the introduction of PCVs (at their DTP3 levels) in these 31 countries would prevent 17*·*6 (95% CrI 11*·*6–23*·*3) million cases, 41 000 (22 400–60 300) deaths, and 2*·*89 (1*·*60–4*·*22) million DALYs.

**Figure 2:**
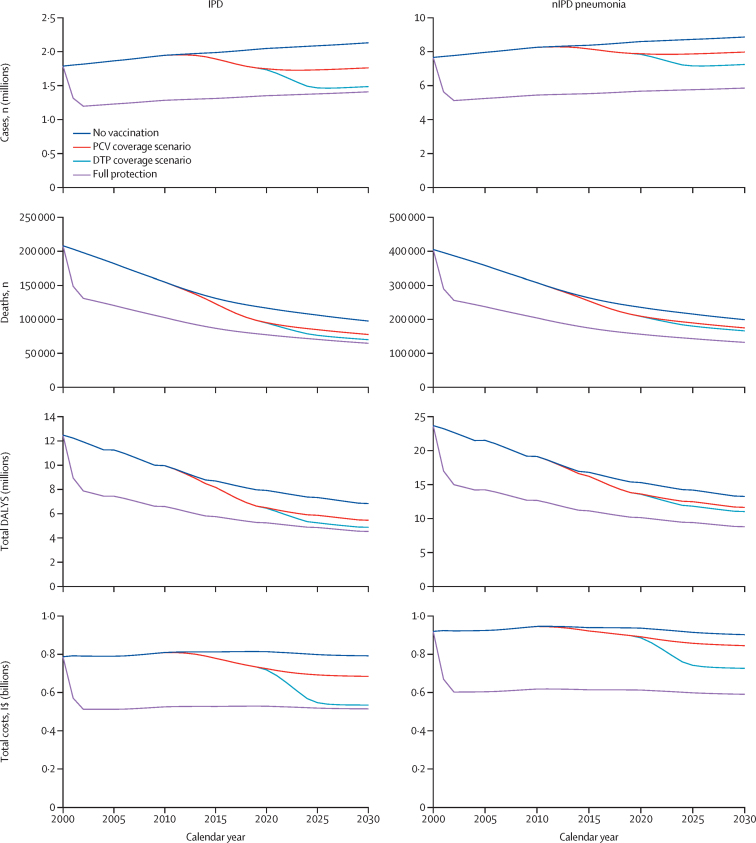
Comparison of outcomes for 112 countries by diseases (IPD and nIPD pneumonia) across time for different modelling scenarios (undiscounted) PCV coverage scenario used respective year's PCV coverage data from 2000 to 2019 and used 2019 PCV coverage data from 2020 to 2030. DTP coverage scenario used respective year's PCV coverage data from 2000 to 2019 and used 2019 DTP coverage data from 2020 to 2030. Full protection was based on Chen and colleagues using regional incidence risk ratios from Flasche and colleagues (2015).^8^, ^12^ This model assumes full protection with elimination of vaccine serotypes and herd protection. Health-care costs arise from hospitalisation and outpatient visits. DALY=disability-adjusted life year. DTP=diphtheria–tetanus–pertussis. IPD=invasive pneumococcal diseases. I$=international dollars. nIPD=non-invasive pneumococcal diseases. PCV=pneumococcal conjugate vaccine.

Using real-world vaccination data compared with the 2019 model, assuming ideal vaccine coverage and timing, PCV impact on pneumococcal deaths in children younger than 5 years were estimated to be reduced by 5*·*3%, lower than the 32*·*1% from the 2019 model (figure 2).^8^ Disease outcomes declined gradually following 2014, when 57 (50*·*9%) of the 112 countries began PCV introduction. From 2020 to 2030, using real-world vaccine coverage data compared with the no vaccination scenario, IPD pneumonia deaths in children younger than 5 years were estimated to reduce by 19*·*9% and nIPD pneumonia deaths by 11*·*9%. Increasing vaccine coverage to DTP coverage levels would result in an estimated 25*·*7% reduction for IPD pneumonia deaths and 15*·*2% reduction for nIPD pneumonia deaths.

We also found that the difference between the estimates with our 2019 model was primarily driven by the low PCV coverage and late vaccine introduction in some countries. When we accounted for these two factors in our 2019 model, we found that the vaccine impact on IPD was similar to our current updated model (appendix pp 22–23).

In the absence of vaccination, the health-system costs associated with pneumococcal disease were estimated at I$1·7 billion per annum (discounted). The projected annual vaccination cost for LMICs was $648 million (discounted) using PCV coverage and $4·2 billion (discounted) for full protection coverage. Globally, from the health-system perspective, the incremental cost of PCV vaccination would be $851 (95% CrI 510–1530; discounted) per DALY averted for the PCV coverage scenario compared with $1320 (806–2310; discounted) per DALY averted for the DPT coverage scenario and $642 (523–813; discounted) per DALY averted in the full protection scenario (table 2). Oceania and Africa have the lowest cost per DALY averted, followed by Asia, Latin America and the Caribbean, and Europe. Undiscounted rates are reported in the appendix (p 19). In the PCV coverage versus no vaccination scenario, vaccination remained cost-effective under all one-way changes to key parameters.

**Table 2:** Outcomes compared with no vaccination scenario with incremental cost-effectiveness ratios compared with I$ per DALYs averted over 30 years, from the health-system perspective (discounted)

	**PCV coverage**	**DPT coverage**	**Full protection**
**DALYs averted (millions)**			
Global	23·5 (12·2–35·2)	28·4 (15·5–42·0)	179 (143–212)
Africa	17·2 (6·63–28·1)	18·3 (7·07–29·9)	93·8 (74·5–111)
Asia	5·39 (2·11–8·88)	9·09 (3·55–15·0)	80·0 (64·0–94·5)
Europe	0·0122 (0·00810–0·0176)	0·0183 (0·0118–0·0269)	0·186 (0·133–0·246)
Latin America and the Caribbean	0·851 (0·590–1·14)	0·898 (0·621–1·20)	4·84 (3·67–5·99)
Oceania	0·0518 (0·0170–0·0877)	0·0531 (0·0174–0·0898)	0·615 (0·490–0·738)
**Health-care costs saved, I$ (billions)**			
Global	1·63 (1·12–2·19)	3·16 (2·10–4·28)	15·0 (12·7–17·7)
Africa	0·717 (0·408–1·02)	0·781 (0·446–1·11)	2·36 (2·01–2·78)
Asia	0·612 (0·330–0·908)	2·04 (1·13–2·94)	11·5 (9·79–13·4)
Europe	0·00286 (0·00167–0·00461)	0·0130 (0·00945–0·0174)	0·0937 (0·0747–0·117)
Latin America and the Caribbean	0·289 (0·198–0·398)	0·318 (0·219–0·437)	1·05 (0·768–1·38)
Oceania	0·00769 (0·00304–0·0137)	0·00808 (0·00320–0·0143)	0·0441 (0·0295–0·0627)
**Vaccination cost, I$ (billions)**			
Global	20·1	38·1	129
Africa	10·6	13·0	33·5
Asia	6·92	22·1	87·1
Europe	0·0629	0·292	1·28
Latin America and the Caribbean	2·48	2·75	6·72
Oceania	0·03117	0·0364	0·131
**Incremental cost-effectiveness ratios, cost (I$) per DALY adverted**			
Global	851 (510–1530)	1320 (806–2310)	642 (523–813)
Africa	642 (341–1530)	742 (395–1760)	336 (277–423)
Asia	1410 (676–3110)	2650 (1270–5870)	957 (780–1210)
Europe	5140 (3320–7610)	15 900 (10200–23700)	6570 (4740–9100)
Latin America and the Caribbean	2650 (1830–3850)	2800 (1930–4070)	1200 (892–1620)
Oceania	549 (205–1610)	629 (245–1820)	145 (93–208)

Data are in mean (95% credible interval). DALY=disability-adjusted life year. DTP=diphtheria–tetanus–pertussis. I$=international $. PCV=pneumococcal conjugate vaccine.

**Figure 3:**
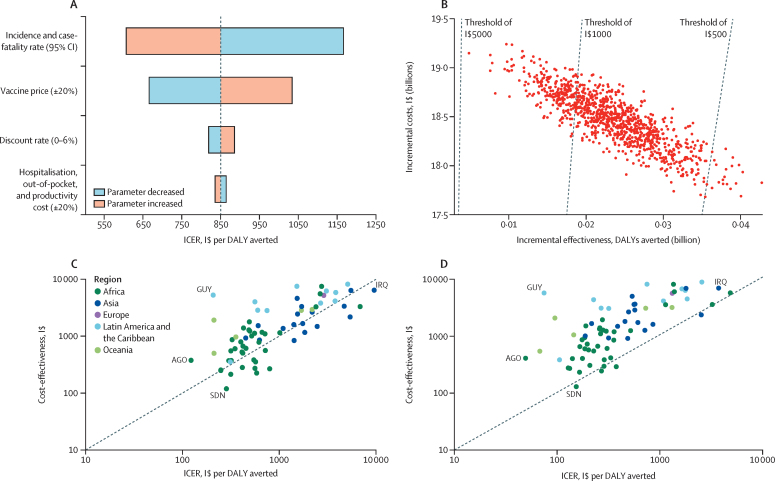
Results of one-way parameter scenario, probabilistic sensitivity analyses, and cost-effectiveness (A) One-way parameter scenario. (B) Probabilistic sensitivity analyses. (C) Cost-effectiveness threshold for PCV coverage scenario. (D) Cost-effectiveness threshold for full coverage scenario. AGO=Angola. DALY=disability-adjusted life year. Africa region includes Algeria, Angola, Benin, Burkina Faso, Burundi, Cabo Verde, Cameroon, Central African Republic, Chad, Comoros, DR Congo, Congo, Côte d’Ivoire, Djibouti, Egypt, Eritrea, Eswatini, Ethiopia, The Gambia, Ghana, Guinea, Guinea-Bissau, Kenya, Lesotho, Liberia, Madagascar, Malawi, Mali, Mauritania, Morocco, Mozambique, Namibia, Niger, Nigeria, Rwanda, São Tomé and Príncipe, Senegal, Sierra Leone, Somalia, South Africa, South Sudan, Sudan, Tanzania, Togo, Tunisia, Uganda, Zambia, and Zimbabwe. Asia region includes Afghanistan, Armenia, Azerbaijan, Bangladesh, Bhutan, Cambodia, China, Georgia, India, Indonesia, Iran, Iraq, Jordan, North Korea, Kyrgyzstan, Laos, Mongolia, Myanmar, Nepal, Pakistan, occupied Palestinian territory, Philippines, Sri Lanka, Syria, Tajikistan, Thailand, Timor-Leste, Turkmenistan, Uzbekistan, Viet Nam, and Yemen. Europe region includes Albania, Belarus, Bosnia and Herzegovina, Kosovo, North Macedonia, Moldova, Serbia, and Ukraine. Latin America region includes Belize, Bolivia, Colombia, Cuba, Ecuador, El Salvador, Guatemala, Guyana, Haiti, Honduras, Jamaica, Nicaragua, Paraguay, Peru, and Venezuela. Oceania region includes Fiji, Kiribati, Marshall Islands, Federated States of Micronesia, Papua New Guinea, Samoa, Solomon Islands, Tonga, Tuvalu, and Vanuatu. GUY=Guyana. ICER=incremental cost-effectiveness ratio. IRQ=Iraq. I$=international dollars. PCV=pneumococcal conjugate vaccine. SDN=Sudan.

The results were most sensitive to variations in disease incidence and mortality parameters (figure 3A). A 20% variation in vaccine price led to a 21*·*7% change in the ICER in either direction. In the probabilistic sensitivity analysis (figure 3B), 100% of simulations resulted in a positive ICER, indicating that, on a global scale, PCV vaccination is associated with an increased reduction of DALYs at higher costs (top right quadrant). PCV introduction was cost-effective in 100% of simulations using a threshold of I$5000 per DALY averted and in 77*·*8% of simulations using a willingness-to-pay threshold of $1000 per DALY averted (figure 3B). However, with a more stringent willingness-to-pay threshold of $500 per DALY averted, PCV vaccination was cost-effective in only 2·0% of simulations (figure 3B). Among the 81 countries with PCV coverage, 72 (88*·*9%) had a cost-effectiveness threshold per life year.^31^ Despite the low PCV coverage, PCV introduction was cost-effective in 54 (75·0%) of the 72 countries from 2000 to 2030, using their respective cost-effectiveness thresholds per life-year (figure 3C). If PCV coverage were increased to DTP coverage levels, PCVs would be cost-effective in one additional country, resulting in 55 countries (76·4%) being cost-effective. For the full coverage scenario, PCVs would be cost-effective in 67 countries (93·1%) using countries’ cost-effectiveness thresholds per life-year (figure 3D).

## Discussion

Our analysis updates our 2019 model^8^ to re-evaluate potential PCV impact using emerging post-vaccination evidence from LMICs. We improved our data sources for several parameters (eg, yearly country-specific vaccine coverage and demographic inputs), we received input from an expert panel, and we incorporated vaccine coverage data.^10^ This update has enabled us to better represent the impact of PCVs in LMICs. In addition, in this model, we incorporated an improved understanding of vaccine impact in LMICs, including the time required to reach post-vaccination equilibrium for vaccine-type carriage, the country-specific average coverage in children younger than 5 years required to fully eliminate vaccine-type *S pneumoniae*, and a differentiated PCV impact for nIPD pneumonia. Unlike other multi-country models, our analysis incorporates indirect effects (herd protection and serotype replacements), assuming that 100% serotype replacement would occur with the introduction of PCVs.

Overall, we found that PCV introduction from 2000 to 2030 has the potential to avert 131 (95% Crl 89*·*0–172) million cases, 697 000 (359 000–1 040 000) deaths, and 46*·*0 (24*·*0–68*·*9) million DALYs in 112 countries. Due to low PCV coverage, the current findings suggest a 5% reduction in pneumococcal deaths among children younger than 5 years, as opposed to the previously projected 34% reduction using full vaccine coverage,^8^ given high coverage, good timeliness, and rapid vaccine introductions globally (figure 2). The 5% reduction is likely an underestimate as PCVs have broader potential benefits in reducing cases, deaths, and DALYs across older age groups. Unlike our previous estimates, our updated estimates use actual PCV coverage in countries (appendix p 4) rather than assuming full PCV coverage, leading to the complete elimination of vaccine types.^8^ This assumption was influential because PCV coverage is lower in LMICs than in high-income countries. Nevertheless, despite the low PCV coverage, vaccination remains cost-effective in 75·0% of countries with ICERs less than their cost-effectiveness thresholds per life year.^31^ The global ICER was estimated to be I$851 per DALY averted. However, in the full protection scenario, the ICER will drop to $642 per DALY averted due to the huge reduction in DALYs across 30 years of protection. Our results are similar to a 2019 systematic review,^32^ in which the ICER in middle-income countries falls in the top right, with I$1085 per DALY averted, where PCV introduction is more costly but more effective than no vaccination. Our updated model also accounted for declining mortality due to improved living standards.^10^ However, delays in vaccine introduction and low PCV coverage continue to cost many children's lives.

Despite the improvements in our model, our study has some limitations. First, our study only focused on the effect of the vaccination on children younger than 5 years. However, vaccinating young children might give indirect protection to other age groups, although this effect is less well documented in LMICs than in high-income countries.^33^ As we only modelled the impact of PCVs on children younger than 5 years, this is likely to underestimate the full impact of PCVs through herd protection, which might have broader potential benefits in reducing cases, deaths, and DALYs among older age groups. We also postulate that the maximum impact of PCVs was achieved at coverage levels of 82*·*1%. This assumes that indirect vaccine effects achieve (near) elimination of vaccine serotypes at this coverage level.

In the updated model, the proportion of IPD pneumonia was estimated to be 14*·*8% of all pneumonia cases using a study conducted in children younger than 5 years in The Gambia.^34^ Although it might not reflect the disease profile of all the other LMICs, it was the best evidence we could identify. Next, our analysis was constrained by the limitation that we assumed a uniform coverage for each country without accounting for potential regional and demographic variations within these countries (eg, urban *vs* rural). In addition, we assumed a constant IRR for countries within the same WHO regions due to the scarcity of country-level IRR estimates. Moreover, our disease burden estimates rely on the syntheses of multiple studies in different regions.^2^, ^35^ Studies in LMICs might underestimate burden (especially for meningitis and NPNM) due to differences in health-care access, availability of antibiotics, and diagnostic procedures. This potential underestimation in burden might imply that the overall benefit of PCVs is greater than we estimated, particularly in LMICs. Additionally, most countries do not have an explicit cost-effectiveness threshold. We used country cost-effectiveness thresholds estimated in a global econometric analysis.^31^ These proposed thresholds are not necessarily valid for country-level decision making, where local thresholds should be used in the context of a deliberative process, considering other factors such as affordability.

Accurately quantifying the potential benefits and long-term impact of PCV introduction on deaths and diseases averted is important. As more countries are self-financing their PCV vaccination programmes, they require detailed information on expected benefits to justify the investment. In summary, introducing PCVs will substantially reduce the burden of pneumococcal infections in LMICs, resulting in lives saved and disease averted. The inclusion of real-world evidence revealed that the impact of PCVs is still substantial but lower than our previous findings due to lower PCV coverage. Our findings highlight the importance of rapid PCV scale-up to high coverage to achieve maximum vaccine impact. Nevertheless, the current reduction is still substantial in absolute terms, even considering the longer time taken to eliminate vaccine-type carriage and the lowered PCV impact that recent data from LMICs after vaccine introduction have shown. Our results provide strong evidence for the introduction of PCVs and highlight the need for countries with low coverage to increase their coverage. Our findings also suggest that these countries will substantially reduce the burden of diseases and deaths by introducing PCVs into their infant immunisation programmes and by improving PCV coverage.

### Contributors

### Data sharing

We used publicly available data for all analyses and the data can be obtained without restrictions, with the sources fully referenced in the text.

## Declaration of interests
