# Supplementary material for: Re-evaluating the impact and cost-effectiveness of pneumococcal conjugate vaccine introduction in 112 low-income and middle-income countries in children younger than 5 years: a modelling study
Source: Lancet Glob Health. 2024 Aug 14;12(9):e1485–97. doi: 10.1016/S2214-109X(24)00232-8 (PMC11345449; doi:10.1016/S2214-109X(24)00232-8)
Supplement: Supplementary appendix [file mmc1.pdf]

THE LANCET
Global Health

Supplementary appendix

This appendix formed part of the original submission and has been peer reviewed. We post it as supplied by the authors.

Supplement to: Chen C, Ang G, Akksilp K, et al. Re-evaluating the impact and cost-effectiveness of pneumococcal conjugate vaccine introduction in 112 low-income and middle-income countries in children younger than 5 years: a modelling study. *Lancet Glob Health* 2024; **12:** e1485–97.

Supplementary appendix

Table of Contents

1.1 Study design 3

1.2 Vaccine coverage 3

1.3 Updated ecological vaccine impact 6

1.3.1 Time taken to the near elimination of VT IPD 6

1.3.2 Vaccine coverage required to reach full vaccine impact 8

1.3.3 Invasive pneumococcal diseases (IPD) 9

1.3.4 Differentiated PCV impact on nIPD pneumonia from experts 9

2 MODEL PARAMETERS ***10***

2.1 PCV introduction details 10

2.2 Disease burden (incidence, severity, and mortality) 13

2.3 DALYs weights for each disease 18

2.4 Probabilistic analysis 18

2.5 Healthcare cost 20

2.6 Incremental cost-effectiveness ratios (ICERs) 22

3. COMPARISONS WITH PREVIOUS MODELS ***24***

REFERENCES ***33***

1 STUDY DESIGN AND MODEL STRUCTURE

1.1 Study design

This model is a re-evaluation of the epidemiological impact of pneumococcal conjugate vaccine (PCV) on children under-5 by adapting the combination of two existing models from Chen et al.[1] and García et al. (which presents the linkage of the ecological model from Chen et al.[1] and the UNIVAC model from García et al.[2,3]) using a decision tree model (**Figure 1a**).

1.2 Vaccine coverage

In total, we tracked 30 birth cohorts of children under-5 between 2000 and 2030 in 112 low- and middle-income countries, including 73 Gavi countries. Accounting for vaccine introduction, estimates in the no vaccination scenario were multiplied by the predicted IRRs from the pseudo-dynamic model adjusted for the abovementioned characteristics to reflect the burden of diseases after PCV introduction. The impact of PCV introduction was estimated by multiplying the predicted incidence risk ratio (IRR) from our 2019 model,[1] to disease estimates from the literature.[4] The IRR was adjusted with WHO-UNICEF vaccine coverage. We used country-specific annual vaccination coverage data from WHO-UNICEF[5] up to 2019. We then compared the estimates from the no vaccination and vaccination scenario.

We present the under-5 vaccine coverage based on two scenarios (**Figure 1b**). The overall line is weighted by population sizes of countries. For the first scenario, national-level PCV coverage from 2000 to 2019 was obtained from WHO-UNICEF Estimates of National Immunization Coverage (WUENIC)[5] and assumed to stay the same for the subsequent years (2020 to 2030). By 2014, at least half the countries had introduced PCV. For the second scenario, from 2000 to 2019, similar to the first scenario, national-level PCV coverage estimates up to 2019 were obtained from WUENIC.[5] However, from 2020 to 2030, we assumed that countries' PCV coverage in 2020 to 2030 would increase to their respective countries' 2019 diphtheria-tetanus-pertussis (DTP) coverage levels, obtained from WUENIC.[6]

Figure 1a Disease pathways for a birth cohort in the vaccination and no vaccination scenario

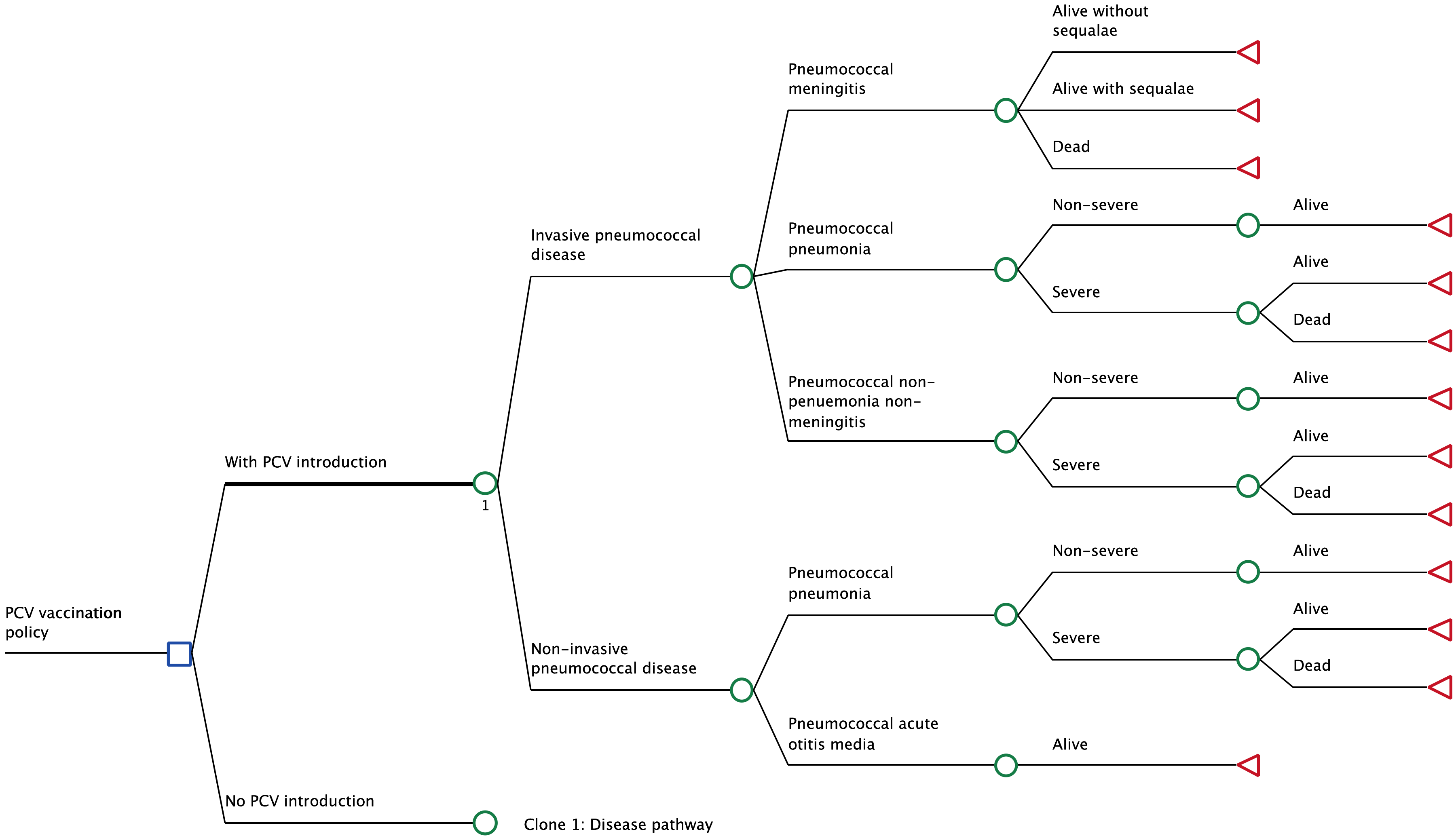

PCV: pneumococcal conjugate vaccine
Clone 1 Disease pathway: repeats the same pathway as the PCV introduction but with different transition probabilities.

Figure 1b Under-5 Coverage for PCV (2000 to 2030) and DTP (2020 to 2030)

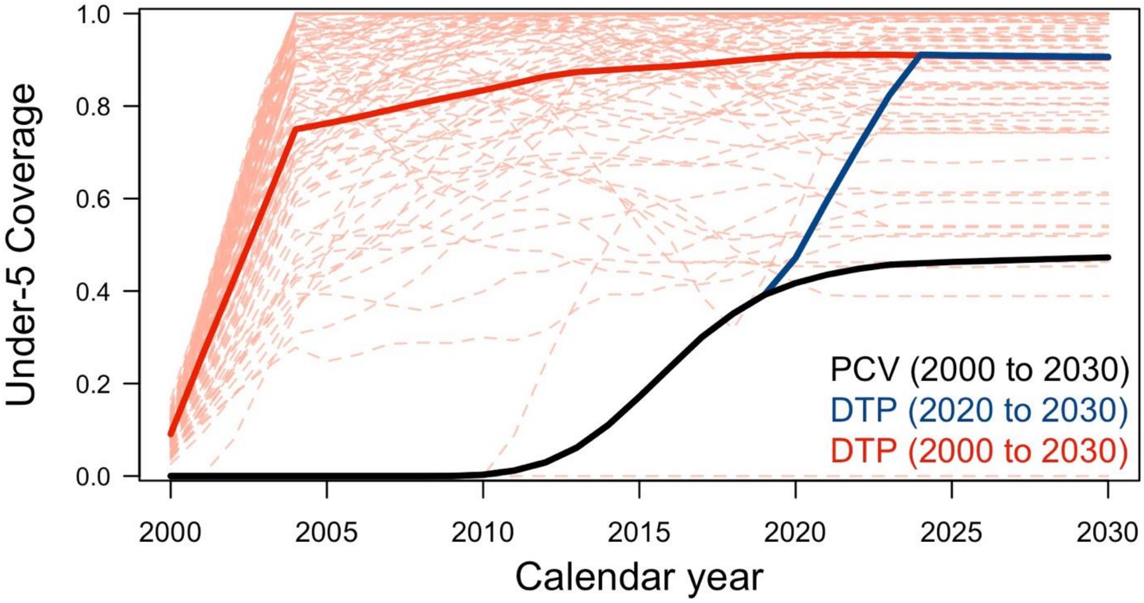

The thick solid lines are the mean under-5 coverage of the countries. The dotted lines are the country-specific under-5 coverage. We assumed that the under-1 coverage is constant from 2019; thus, the under-5 will be constant from 2024 (see the next paragraph).
DTP: diphtheria-tetanus-pertussis; PCV: pneumococcal conjugate vaccine.

Using Bosnia and Herzegovina as an example, we illustrate why under-5 coverage will only be constant from 2024. Bosnia and Herzegovina was one of the 31 countries without PCV in 2019.

Table 1. Bosnia and Herzegovina under -1 and under-5 vaccine coverage for the scenario with PCV introduction at DTP coverage in 31 countries without PCV in 2019

	**2016**	**2017**	**2018**	**2019**	**2020**	**2021**	**2022**	**2023**	**2024**	**2025**	**…**	**2030**
**Under-1**	0[a]	0[a]	0[a]	0[a]	0.81[b]	0.81[b]	0.81[b]	0.81[b]	0.81[b]	0.81[b]	…	0.81[b]
**Under-5**	0	0	0	0	0.065[c]	0.24	0.41	0.60	0.78	0.78	…	0.78

a Bosnia and Herzegovina had yet to introduce PCV as of 2019.
b We assume the PCV coverage from 2020 to 2030 is Bosnia and Herzegovina’s 2019 DTP vaccine coverage.
c The 2020 under-5 coverage is computed using the under-1 coverage from 2016 to 2020.

1.3 Updated ecological vaccine impact

A recent systematic review conducted amongst under-5 in low- and middle-income countries found that although the introduction of PCV has led to a decrease in the prevalence of vaccine-type (VT) carriage, the prevalence of non-vaccine-type (NVT) carriage has increased due to serotype replacement.[7] The authors found that the carriage of NVTs could increase up to 74.1% post-vaccination.

Countries with high under-5 coverage were assumed to receive the full impact, but we accounted for the time taken to eliminate VT IPD and the vaccine coverage required to reach full impact. The updated model is an extension of Chen et al.[1] model by incorporating three new model characteristics as explained in the main paper. First, the previous model assumed that the time taken to the near elimination of VT IPD was two years, but recent evidence suggests this may be closer to five years.[8] Second, 100% PCV coverage was assumed as only some LMICs had post-introduction coverage data. Third, with this high PCV programme coverage, countries were assumed to reach equilibrium in VT carriage with existing PCV coverage and herd effects.[1] Lastly, as the evidence of the impact of PCV against nIPD is unclear,[9] the previous model assumed the same PCV impact for non-invasive pneumococcal disease (nIPD) and IPD pneumonia.[1] However, it is likely that PCV effectiveness differs against disease with varying levels of invasiveness. Thus, this study updates our previous estimates by combining a country-specific model with emerging evidence on the real-world impact of PCV vaccine coverage. Below is a detailed description of the extension not explained in the main paper.

1.3.1 Time taken to the near elimination of VT IPD

A recent systematic review and meta-analysis had shown that the time taken to the near-elimination of vaccine-type IPD could be longer than the previously assumed two years.[8] Using the definition of near-elimination of vaccine-type IPD as the time required to reduce corresponding disease by 90%, the authors found that for children under-5, the mean period to attain 90% reduction for all PCV7 serotypes was 4·6 years (95% CrI: 3·9-6·0). Hence, we updated the model with the year-on-year reductions in vaccine-type IPD, until near elimination of vaccine-type IPD was achieved. Using results from a systematic review and meta-analysis by Shiri et al.[8] specific for children under five and PCV7, we fitted a log-linear model to interpolate the percentage reduction in the incidence of IPD due to PCV7 serotypes for one to five years after vaccine introduction. As defined by the study, near elimination of IPD is the time required to reduce disease by 90%. Assuming 0% elimination of vaccine serotypes (VT) IPD upon vaccine introduction, and the findings from the meta-analysis that 1.2 years and 4.6 years are required to reduce VT IPD by 50% and 90% respectively, we estimated that the percentage elimination of VT IPD for 1 to 5 years post-vaccine introduction was **42%, 64%, 78%, 87% and 92%** (**Figure 2**)**.** We referenced evidence from PCV7 because the vaccine impact used was estimated by Flasche et al.[10] was modelled using PCV7. Due to the lack of evidence, we assumed that IPD and nIPD take the same time to reach the near elimination of VT disease.

Figure 2 Years required to eliminate PCV7 serotypes

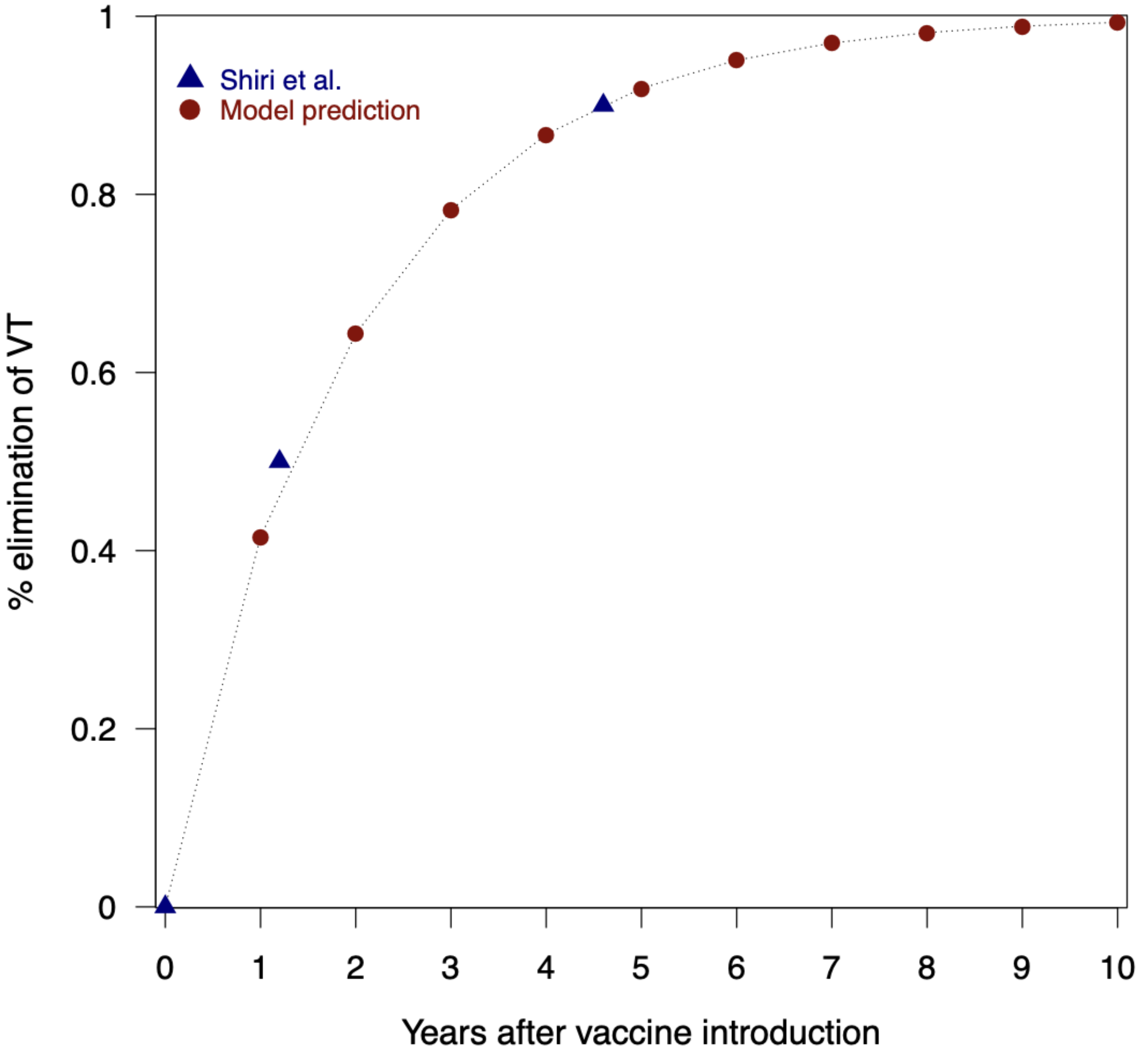

1.3.2 Vaccine coverage required to reach full vaccine impact

To account for each country's vaccination coverage, we started with annual average PCV coverage in children under-5 using vaccine coverage for three doses, accounting for country-specific vaccine introduction year.[2,11] We estimated the average coverage achieved for each age category (under 1, 1 to 2, 2 to 3, 3 to 4 and 4 to 5 years) from UNIVAC's timeliness by the week of age.[12] We fitted a linear regression model to estimate the vaccine coverage needed for IPD to be reduced by 90%, as defined by the authors, to achieve near vaccine-type IPD elimination.[8] The pseudo-dynamic model assumed that vaccine-type carriage would be eliminated at high PCV coverage. We used published studies to determine that 82·1% (95%CI: 46.7-117) PCV coverage was needed to reduce vaccine-type IPD by 90%. Thus, we assumed that approximately 82% of vaccine coverage is required to reach full impact. This coverage value was estimated using evidence from four studies [13-16] in Kenya, Gambia, Israel, and the United Kingdom about the maximum VT IPD reduction following vaccine introduction. For each study, we identified the PCV coverage, and the reduction in pneumococcal disease. These results were then fitted into a linear regression and used to estimate the mean coverage required to achieve a 90% reduction (**Figure 3**). We did not account for different vaccine impacts from receiving one or two doses, or waning vaccine protection over time.

Figure 3 Pneumococcal conjugate vaccine coverage to eliminate vaccine-type (VT) carriage

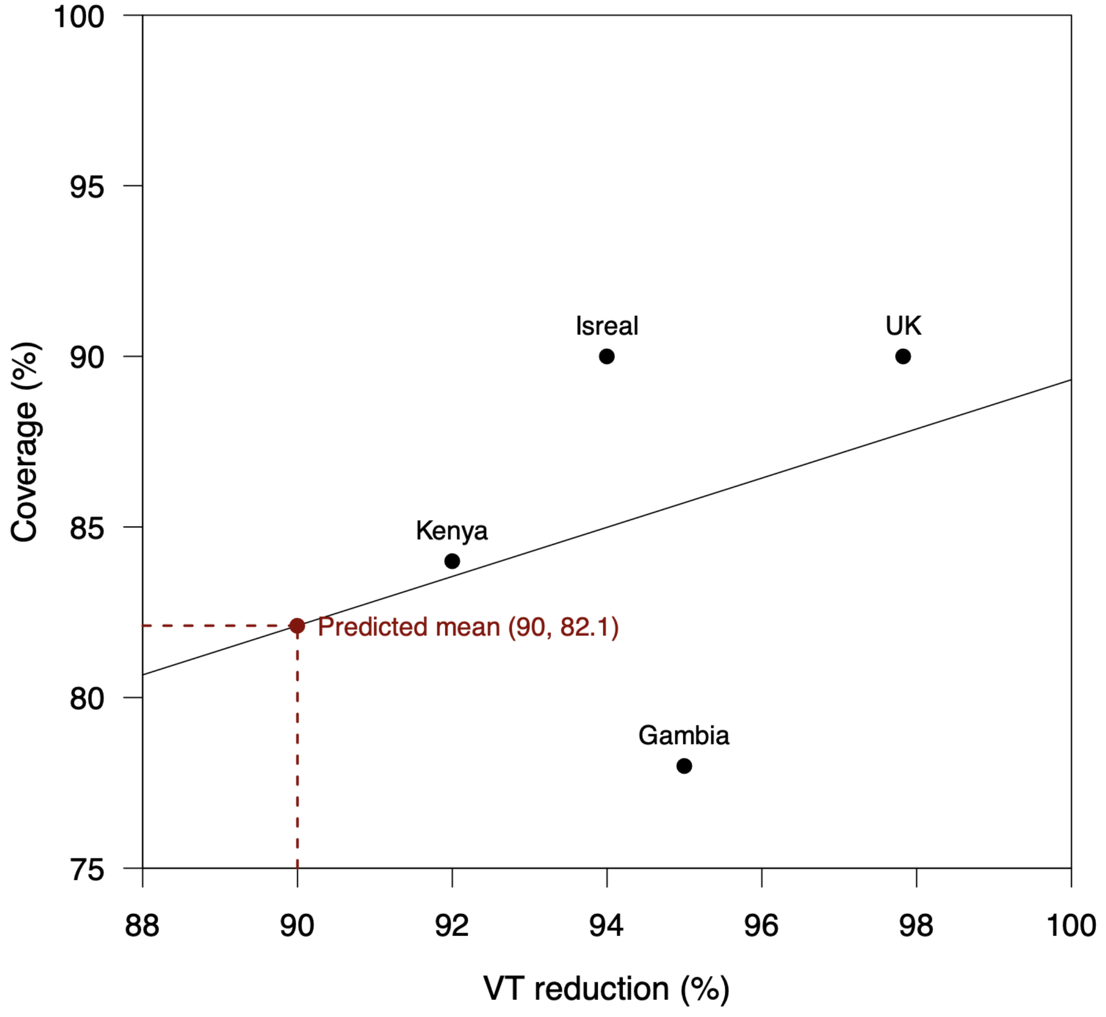

1.3.3 Invasive pneumococcal diseases (IPD)

In our study, meningitis and NPNM were classified as invasive pneumococcal diseases (IPD). For pneumococcal pneumonia, based on a study conducted in The Gambia [17] for children, we assumed that 14.8% of the disease burden was IPD. This was estimated by assuming that 13.35% [4] of clinical pneumonia in the placebo group (n = 2284, Table 3 [17]) was attributable to pneumococcus. The proportion of IPD pneumococcal pneumonia can thus be estimated by taking a ratio of the number of IPD radiological pneumonia (n = 45, Table 4 [17]) to the number of pneumococcal clinical pneumonia cases (n = 2284 x 0.1335). This assumption was different from the earlier model by Chen et al.[1], where they assumed 24.8% of pneumonia to be bacteraemic based evidence amongst adults[18] due to the lack of evidence.

1.3.4 Differentiated PCV impact on nIPD pneumonia from experts

A panel of experts on pneumococcal epidemiology and microbiology was assembled to input on the parameters and assumptions to be used in the model. A meeting was held over Zoom on 3 August 2021 for members able to join; others were consulted by email.

Expert	Affiliation	Attended the meeting
**Billy Quilty**	Research Fellow,<br>London School of Hygiene & Tropical Medicine (LSHTM)	Yes
**Brenda Kwambana-Adams**	Senior Research Fellow,<br>University College London, Division of Infection & Immunity	Yes
**Dan Weinberger**	Associate Professor in Epidemiology of Microbial Diseases,<br>Yale School of Public Health	Yes
**Stefan Flasche**	Professor of Vaccine Epidemiology,<br>London School of Hygiene & Tropical Medicine (LSHTM)	No

The meeting started with a summary presentation by Mark Jit and Cynthia Chen detailing the model's motivation, methods, and assumptions. Mark Jit also presented the latest emerging real-world evidence from post-PCV introduction studies from Kenya (Kilifi), Gambia, Israel, England, Lao PDR, and Mongolia. As each country has different vaccine coverage and impact, this was followed by a discussion with the following questions listed below. Finally, the scholars debated on the best assumption for a global analysis based on these emerging studies.

1. What is the percentage coverage to achieve herd immunity/elimination of vaccine types?
2. How long does it take for the vaccine to reach its maximum impact?
3. What should be the vaccine impact on pneumococcal AOM?
4. What should be the vaccine impact on Pneumonia, Meningitis, NPNM?
5. What could be a suitable case definition for IPD?
6. What could be a suitable case definition for non-invasive pneumococcal pneumonia?
7. What could be a suitable case definition for pneumococcal AOM?

The panellists continued to provide input during and after the meeting and also shared a slide on VE against AOM compared to IPD and pneumonia. The team continues to follow up on the suggestions from the panellist. The team continued to exchange follow-up discussions until 7th December 2021 via email, where panellists provided inputs and shared published literature relevant to our study. After this, the model parameters were confirmed. Finally, the vaccine impact assumptions were also confirmed with Anthony Scott via email.

Based on a study conducted in Gambia,[17] we assumed that 14·8% of pneumonia was bacteraemic, and the rest was nIPD pneumonia. This was estimated by assuming that 13·4% of clinical pneumonia in the placebo group was attributable to pneumococcus.[4] The panel of pneumococcal experts was presented with questions about PCV effectiveness and disease endpoints and agreed on the assumption for PCV impact on nIPD to be 60% of the PCV impact on IPD. This 60% was estimated by assuming the midpoint for the vaccine efficacy (VE) against VT carriage and IPD as a proxy for the VE against nIPD. We assumed the lower bound of the VE against carriage for hospitalised children as the VE against carriage at 11.4% (results from Table 3: 39.1%; 95% CI: 11.4-58.1) from the study conducted by Chan et al.[19] in Mongolia. As the same study did not provide VE against IPD, we assumed VE against IPD from a Cochrane Review by Lucero et al.[20] at 58% (95% CI: 29-75). Taking the midpoint of the two VE gives 34.7% for the VE against nIPD. This is about 60% of the VE against IPD.
Furthermore, we assumed that vaccine impact on pneumococcal AOM was the expected number of AOM outcomes in a vaccination scenario multiplied by the expected outcomes with the expected PCV coverage and vaccine efficacy for each vaccination dose. Vaccine efficacy for pneumococcal acute otitis media was assumed to be 20% based on a Cochrane Review.[21]

2 MODEL PARAMETERS

The model evaluated the impact of PCV in a total of 112 low- and middle-income countries, including 73 Gavi countries. Among the 73 Gavi countries, Mongolia was the first to transit out of Gavi's support to fully finance their PCV immunisation programmes in 2015,[22] followed by Bhutan, Indonesia, Timor-Leste and Ukraine.[22] These countries were categorised into five of the six UN regions available (Africa, Asia, Europe, Latin America and Oceania). Countries in each region are listed in **Table 2** below together with their respective PCV introduction details.

2.1 PCV introduction details

Among the 81 out of 112 countries with PCV coverage by 2019, the two main PCVs currently used in infant immunisation programmes are PCV10 (n=17) and PCV13 (n=63), with one country using a mix of PCV10 and PCV13 (n=1). The difference in effectiveness between PCV10 and PCV13 is unclear, as earlier studies have also found cross-protection of PCV10 against additional serotypes.[23-25] Most countries adopt a 3-dose schedule, where children receive either three primary doses at two, four, and six months of age, without a booster dose (the 3+0 schedule) or two primary doses at two and four months of age, followed by one booster dose at 12-15 months of age (the 2+1 schedule).[26,27] These countries also have different vaccine coverage.[2,3,11,28] Countries with low or no PCV coverage also tend to have high disease incidence among the under-5.[1,2,29]

Table 2 PCV introduction details by regions and countries

Region	Country	PCV introduction	Introduction year	Gavi	Brand	Schedule
Africa	Algeria	Yes	2016	No	Prevnar (PCV13)	2+1
	Angola	Yes	2013	Yes	Prevnar (PCV13)	3+0
	Benin	Yes	2011	Yes	Prevnar (PCV13)	3+0
	Burkina Faso	Yes	2014	Yes	Prevnar (PCV13)	3+0
	Burundi	Yes	2011	Yes	Prevnar (PCV13)	3+0
	Cabo Verde	No	2024	No	-	-
	Cameroon	Yes	2011	Yes	Prevnar (PCV13)	3+0
	Central African Republic (the)	Yes	2011	Yes	Prevnar (PCV13)	3+0
	Chad	No	2027	Yes	-	-
	Comoros (the)	No	2025	Yes	-	-
	Congo (the Democratic Republic of the)	Yes	2011	Yes	Prevnar (PCV13)	3+0
	Congo (the)	Yes	2012	Yes	Prevnar (PCV13)	3+0
	Côte d'Ivoire	Yes	2014	Yes	Prevnar (PCV13)	3+0
	Djibouti	Yes	2013	Yes	Prevnar (PCV13)	3+0
	Egypt	No	2024	No	-	-
	Eritrea	Yes	2016	Yes	Prevnar (PCV13)	3+0
	Eswatini	Yes	2014	No	Prevnar (PCV13)	3+0
	Ethiopia	Yes	2011	Yes	Synflorix (PCV10)	3+0
	Gambia (the)	Yes	2010	Yes	Prevnar (PCV13)	3+0
	Ghana	Yes	2012	Yes	Prevnar (PCV13)	3+0
	Guinea	No	2027	Yes	-	-
	Guinea-Bissau	Yes	2015	Yes	Prevnar (PCV13)	3+0
	Kenya	Yes	2011	Yes	Synflorix (PCV10)	3+0
	Lesotho	Yes	2015	Yes	Prevnar (PCV13)	3+0
	Liberia	Yes	2014	Yes	Prevnar (PCV13)	3+0
	Madagascar	Yes	2013	Yes	Synflorix (PCV10)	3+0
	Malawi	Yes	2012	Yes	Prevnar (PCV13)	3+0
	Mali	Yes	2011	Yes	Prevnar (PCV13)	3+0
	Mauritania	Yes	2013	Yes	Prevnar (PCV13)	3+0
	Morocco	Yes	2010	No	Synflorix (PCV10)	2+1
	Mozambique	Yes	2013	Yes	Prevnar (PCV13)	3+0
	Namibia	Yes	2015	No	Prevnar (PCV13)	3+0
	Niger	Yes	2014	Yes	Prevnar (PCV13)	3+0
	Nigeria	Yes	2015	Yes	Synflorix (PCV10)	3+0
	Rwanda	Yes	2010	Yes	Prevnar (PCV13)	3+0
	Sao Tome and Principe	Yes	2013	Yes	Prevnar (PCV13)	3+0
	Senegal	Yes	2014	Yes	Prevnar (PCV13)	3+0
	Sierra Leone	Yes	2011	Yes	Prevnar (PCV13)	3+0
	Somalia	No	2027	Yes	-	-
	South Africa	Yes	2009	No	Prevnar (PCV13)	2+1
	South Sudan	No	2027	Yes	-	-
	Sudan	Yes	2013	Yes	Prevnar (PCV13)	3+0
	Tanzania, United Republic	Yes	2013	Yes	Prevnar (PCV13)	3+0
	Togo	Yes	2014	Yes	Prevnar (PCV13)	3+0
	Tunisia	No	2020	No	Synflorix (PCV10)	-
	Uganda	Yes	2014	Yes	Synflorix (PCV10)	3+0
	Zambia	Yes	2014	Yes	Synflorix (PCV10)	3+0
	Zimbabwe	Yes	2012	Yes	Prevnar (PCV13)	3+0
Asia	Afghanistan	Yes	2014	Yes	Prevnar (PCV13)	3+0
	Armenia	Yes	2015	Yes	Prevnar (PCV13)	3+0
	Azerbaijan	Yes	2014	Yes	Prevnar (PCV13)	3+0
	Bangladesh	Yes	2015	Yes	Synflorix (PCV10)	3+0
	Bhutan	Yes	2019	Yes	Prevnar (PCV13)	-
	Cambodia	Yes	2015	Yes	Prevnar (PCV13)	3+0
	China	No	2023	No	-	-

Region	Country	PCV introduction	Introduction year	Gavi	Brand	Schedule
	Georgia	Yes	2015	Yes	Synflorix (PCV10)	2+1
	India	Yes	2018	Yes	PNEUMOSIL (PCV10)	2+1
	Indonesia	Yes	2019	Yes	Prevnar (PCV13)	2+1
	Iran (Islamic Republic)	No	2023	No	-	-
	Iraq	Yes	2017	No	Prevnar (PCV13)	3+0
	Jordan	No	2025	No	-	-
	Korea (the Democratic People's Republic)	No	2024	Yes	-	-
	Kyrgyzstan	Yes	2017	Yes	Prevnar (PCV13)	2+1
	Lao People's Democratic Republic	Yes	2014	Yes	Prevnar (PCV13)	3+0
	Mongolia	Yes	2017	Yes	Prevnar (PCV13)	2+1
	Myanmar	Yes	2016	Yes	Prevnar (PCV13)	3+0
	Nepal	Yes	2015	Yes	Synflorix (PCV10)	2+1
	Pakistan	Yes	2013	Yes	Synflorix (PCV10)	3+0
	Palestine		2012			
	Philippines	Phased	2014	No	Synflorix (PCV10) & Prevnar (PCV13)	3+1
	Sri Lanka	No	2023	Yes	-	-
	Syrian Arab Republic	No	2027	No	-	-
	Tajikistan	No	2024	Yes	-	-
	Thailand	No	2024	No	-	-
	Timor-Leste	No	2026	Yes	-	-
	Turkmenistan	No	2020	No	-	-
	Uzbekistan	Yes	2016	Yes	Prevnar (PCV13)	2+1
	Viet Nam	No	2025	Yes	-	-
	Yemen	Yes	2011	Yes	Prevnar (PCV13)	3+0
**Europe**	Albania	Yes	2011	No	Prevnar (PCV13)	3+0
	Belarus	Risk	2023	No	Synflorix (PCV10)	3+1
	Bosnia and Herzegovina	No	2026	No	-	-
	Kosovo		2018			
	Macedonia (the former Yugoslav Republic)	No	2025	No	-	-
	Moldova (the Republic)	Yes	2013	Yes	Prevnar (PCV13)	2+1
	Serbia	No	2024	No	-	-
	Ukraine	No	2026	Yes	-	-
**Latin America**	Belize	No	2024	No	-	-
	Bolivia (Plurinational State)	Yes	2014	Yes	Prevnar (PCV13)	3+0
	Colombia	Yes	2010	No	Synflorix (PCV10)	2+1
	Cuba	No	2023	Yes	-	-
	Ecuador	Yes	2010	No	Synflorix (PCV10)	3+0
	El Salvador	Yes	2010	No	Synflorix (PCV10)	2+1
	Guatemala	Yes	2013	No	Prevnar (PCV13)	2+1
	Guyana	Yes	2011	Yes	Prevnar (PCV13)	3+0
	Haiti	Yes	2018	Yes	Prevnar (PCV13)	3+0
	Honduras	Yes	2011	Yes	Prevnar (PCV13)	3+0
	Jamaica	Risk	2024	No	Prevnar (PCV13)	3+1
	Nicaragua	Yes	2011	Yes	Prevnar (PCV13)	3+0
	Paraguay	Yes	2012	No	Prevnar (PCV13)	2+1
	Peru	Yes	2009	No	Prevnar (PCV13)	2+1
	Venezuela (Bolivarian Republic)	Yes	2015	No	Prevnar (PCV13)	2+1
**Oceania**	Fiji	Yes	2013	No	Synflorix (PCV10)	3+0
	Kiribati	Yes	2013	Yes	Prevnar (PCV13)	3+0
	Marshall Islands	Yes	2009	No	Prevnar (PCV13)	3+1
	Micronesia (Federated States)	Yes	2010	No	Prevnar (PCV13)	3+1
	Papua New Guinea	Yes	2015	Yes	Prevnar (PCV13)	3+0
	Samoa	No	2026	No	-	-

Region	Country	PCV introduction	Introduction year	Gavi	Brand	Schedule
	Solomon Islands	Yes	2015	Yes	Prevnar (PCV13)	3+0
	Tonga	No	2023	No	-	-
	Tuvalu	No	2025	No	-	-
	Vanuatu	No	2025	No	-	-

2.2 Disease burden (incidence, severity, and mortality)

Disease burden estimates (**Table 3**) were obtained from a global burden study by Wahl et al..[4] The study provides country-specific incidence and mortality rates for pneumococcal pneumonia (severe and non-severe), pneumococcal meningitis and NPNM (severe and non-severe). Based on the World Health Organisation, severe pneumonia is defined as having any general danger signs, chest indrawing (symptom or visual) or stridor in a child with a cough or difficulty breathing.[30] This definition was used instead of the WHO updated definition in 2013[31] as they informed most impact studies from pre-vaccination surveillance. The risk of disabling sequelae from pneumococcal meningitis was obtained from a review study[32] to project the incidence of meningitis sequelae. Furthermore, incidence rates for pneumococcal AOM were obtained from a global systematic review,[33] assuming that 20% of the burden was attributed to *Streptococcus pneumoniae*.[34] For all non-severe diseases and pneumococcal AOM, the duration of illness was assumed to be seven days.[35-37] For severe diseases, we assumed the duration of illness to be ten days [38,39]. Pneumococcal meningitis sequelae were assumed to last 50 years. In addition, only severe diseases and diseases related to meningitis led to mortality as published by Wahl et al..[4] Consistent with UNIVAC, we assumed pneumococcal mortality rates would decrease over time (using the same rate of decrease reported for all-cause under-five deaths) in the absence of vaccination, due to improvements in access to care and living standards.

Table 3 Pre-vaccination incidence and mortality rates per 100,000 population among children under five years of age

Country	Incidence rates (low, high)							Mortality rates (low, high)		
	Pneumonia (non-severe)	Pneumonia (severe)	NPNM (non-severe)	NPNM (severe)	Meningitis	Meningitis sequelae	Acute otitis media	Pneumonia (severe)	NPNM (severe)	Meningitis
Afghanistan	42 (18, 86)	7 (2, 18)	126 (54, 258)	17 (7, 34)	2153 (2017, 2627)	1402 (1050, 1599)	12649 (12513, 12787)	22 (9, 45)	7 (3, 14)	133 (94, 138)
Albania	5 (1, 19)	1 (0, 6)	14 (3, 57)	5 (1, 21)	81 (87, 103)	149 (112, 170)	5884 (5746, 6020)	2 (0, 7)	1 (0, 6)	18 (13, 19)
Algeria	6 (2, 13)	1 (0, 4)	17 (5, 41)	6 (2, 15)	1008 (944, 1229)	651 (488, 743)	8139 (8001, 8275)	2 (1, 5)	2 (1, 5)	22 (15, 22)
Angola	41 (22, 76)	4 (2, 9)	122 (66, 229)	16 (9, 30)	705 (659, 859)	450 (337, 513)	26584 (26446, 26721)	30 (16, 56)	9 (5, 18)	258 (183, 269)
Armenia	8 (3, 19)	2 (1, 6)	25 (9, 58)	9 (4, 22)	81 (87, 103)	149 (112, 170)	8032 (7894, 8170)	3 (1, 6)	2 (1, 6)	10 (7, 10)
Azerbaijan	5 (2, 12)	1 (0, 2)	16 (5, 36)	6 (2, 13)	81 (87, 103)	149 (112, 170)	8032 (7894, 8170)	3 (1, 7)	3 (1, 6)	34 (24, 36)
Bangladesh	7 (2, 17)	1 (0, 3)	22 (7, 50)	8 (2, 19)	1233 (1155, 1504)	798 (598, 910)	12649 (12513, 12787)	5 (1, 11)	4 (1, 9)	33 (23, 34)
Belarus	2 (1, 4)	0 (0, 1)	5 (2, 12)	2 (1, 4)	162 (173, 207)	298 (223, 340)	6576 (6438, 6714)	0 (0, 1)	0 (0, 1)	2 (1, 2)
Belize	12 (5, 27)	3 (1, 10)	36 (16, 81)	13 (6, 31)	747 (699, 911)	479 (359, 546)	9223 (9086, 9361)	3 (1, 6)	2 (1, 5)	10 (7, 11)
Benin	31 (11, 70)	3 (1, 9)	95 (34, 212)	13 (5, 28)	1369 (1281, 1670)	880 (660, 1004)	28546 (28410, 28680)	22 (8, 50)	7 (3, 16)	135 (96, 141)
Bhutan	9 (4, 17)	2 (1, 5)	27 (12, 52)	10 (5, 19)	785 (735, 958)	501 (376, 572)	12649 (12513, 12787)	3 (2, 7)	3 (1, 6)	29 (21, 30)
Bolivia, Plurinational State of	7 (2, 18)	1 (0, 4)	20 (5, 54)	8 (2, 20)	552 (516, 673)	353 (264, 402)	5986 (5850, 6124)	4 (1, 10)	3 (1, 9)	38 (27, 39)
Bosnia and Herzegovina	1 (0, 2)	0 (0, 1)	2 (1, 5)	1 (0, 2)	81 (87, 103)	149 (112, 170)	5884 (5746, 6020)	0 (0, 0)	0 (0, 0)	1 (1, 1)
Burkina Faso	45 (24, 83)	9 (4, 20)	137 (73, 251)	18 (10, 33)	1223 (1145, 1492)	793 (594, 904)	28546 (28410, 28680)	21 (11, 38)	7 (3, 12)	109 (77, 114)
Burundi	61 (31, 116)	11 (4, 27)	185 (93, 351)	25 (12, 47)	1029 (964, 1256)	665 (498, 759)	15019 (14881, 15157)	30 (15, 57)	9 (5, 18)	125 (89, 131)
Cabo Verde	33 (21, 45)	10 (5, 18)	100 (63, 135)	38 (24, 51)	988 (925, 1205)	637 (477, 726)	28546 (28410, 28680)	4 (3, 6)	4 (2, 5)	24 (17, 25)
Cambodia	11 (3, 26)	2 (1, 7)	32 (10, 79)	12 (4, 30)	1134 (1062, 1384)	735 (550, 838)	8150 (8012, 8286)	4 (1, 11)	4 (1, 10)	29 (21, 30)
Cameroon	26 (9, 59)	2 (1, 7)	78 (27, 178)	10 (4, 24)	1070 (1002, 1305)	687 (515, 784)	28546 (28410, 28680)	19 (7, 43)	6 (2, 14)	130 (92, 136)
Central African Republic	50 (17, 113)	5 (1, 14)	150 (52, 340)	20 (7, 45)	1013 (948, 1236)	653 (489, 745)	26584 (26446, 26721)	36 (13, 82)	11 (4, 26)	167 (118, 174)
Chad	56 (21, 123)	6 (2, 17)	168 (63, 371)	22 (8, 49)	1075 (1005, 1310)	682 (511, 778)	28546 (28410, 28680)	38 (14, 85)	12 (5, 27)	245 (174, 256)
China	11 (6, 15)	3 (2, 6)	32 (19, 44)	12 (7, 17)	379 (354, 462)	240 (180, 274)	5343 (5207, 5481)	1 (1, 2)	1 (1, 2)	6 (5, 7)
Colombia	5 (1, 15)	1 (0, 4)	15 (3, 46)	6 (1, 17)	557 (521, 679)	356 (267, 407)	6491 (6355, 6629)	2 (0, 6)	2 (0, 5)	15 (11, 16)
Comoros	17 (5, 40)	2 (0, 6)	52 (16, 120)	20 (6, 45)	1338 (1253, 1632)	867 (649, 988)	15019 (14881, 15157)	12 (4, 27)	10 (3, 24)	66 (47, 68)
Congo	11 (4, 24)	2 (1, 5)	34 (13, 73)	13 (5, 27)	1004 (940, 1225)	649 (487, 741)	26584 (26446, 26721)	6 (2, 13)	5 (2, 12)	50 (36, 52)
Cote d'Ivoire	21 (8, 45)	2 (1, 7)	62 (24, 136)	8 (3, 18)	578 (541, 705)	369 (276, 421)	28546 (28410, 28680)	14 (5, 30)	4 (2, 9)	95 (68, 99)
Cuba	9 (4, 18)	2 (1, 7)	26 (11, 55)	10 (4, 21)	81 (87, 103)	149 (112, 170)	9223 (9086, 9361)	2 (1, 3)	1 (1, 3)	4 (3, 4)
Korea, Democratic People's Republic of	13 (3, 32)	3 (1, 9)	38 (11, 97)	14 (4, 36)	656 (613, 799)	417 (313, 476)	5343 (5207, 5481)	5 (1, 12)	4 (1, 11)	27 (19, 28)

Country	Incidence rates (low, high)							Mortality rates (low, high)		
	Pneumonia (non-severe)	Pneumonia (severe)	NPNM (non-severe)	NPNM (severe)	Meningitis	Meningitis sequelae	Acute otitis media	Pneumonia (severe)	NPNM (severe)	Meningitis
**Congo, the Democratic Republic of the**	44 (17, 95)	5 (2, 15)	133 (52, 288)	18 (7, 38)	1236 (1158, 1508)	802 (601, 915)	26584 (26446, 26721)	28 (11, 62)	9 (3, 19)	132 (94, 138)
**Djibouti**	50 (25, 75)	13 (5, 26)	150 (76, 226)	56 (29, 85)	869 (813, 1060)	560 (420, 639)	15019 (14881, 15157)	12 (6, 18)	11 (5, 16)	63 (45, 66)
**Ecuador**	45 (22, 70)	14 (5, 27)	136 (67, 210)	51 (25, 79)	566 (530, 690)	362 (271, 413)	5986 (5850, 6124)	6 (3, 9)	5 (3, 8)	25 (17, 26)
**Egypt**	4 (1, 9)	1 (0, 3)	12 (4, 28)	4 (1, 10)	522 (489, 637)	334 (250, 381)	8139 (8001, 8275)	1 (0, 3)	1 (0, 3)	23 (16, 24)
**El Salvador**	9 (3, 23)	2 (1, 7)	26 (8, 70)	10 (3, 26)	745 (697, 908)	476 (357, 543)	6491 (6355, 6629)	2 (1, 7)	2 (1, 6)	20 (14, 21)
**Eritrea**	20 (7, 45)	3 (1, 8)	61 (20, 135)	23 (8, 51)	1076 (1007, 1312)	692 (518, 789)	15019 (14881, 15157)	12 (4, 27)	11 (4, 24)	62 (44, 65)
**Ethiopia**	18 (5, 42)	2 (0, 6)	54 (16, 126)	20 (6, 48)	1084 (1015, 1322)	697 (522, 795)	15019 (14881, 15157)	13 (4, 29)	11 (3, 26)	71 (51, 74)
**Fiji**	46 (20, 81)	14 (5, 32)	138 (62, 245)	52 (23, 92)	726 (679, 885)	464 (348, 529)	21500 (21362, 21637)	6 (3, 10)	5 (2, 9)	25 (18, 26)
**Gambia**	37 (16, 73)	7 (2, 18)	111 (48, 221)	42 (18, 83)	1080 (1011, 1318)	695 (520, 792)	28546 (28410, 28680)	16 (7, 33)	15 (6, 29)	88 (62, 92)
**Georgia**	2 (0, 7)	0 (0, 1)	7 (1, 20)	3 (0, 8)	81 (87, 103)	149 (112, 170)	8032 (7894, 8170)	2 (0, 5)	1 (0, 4)	6 (4, 6)
**Ghana**	16 (6, 34)	3 (1, 7)	48 (18, 104)	18 (7, 39)	852 (797, 1039)	547 (410, 624)	28546 (28410, 28680)	9 (3, 19)	8 (3, 17)	64 (46, 67)
**Guatemala**	8 (2, 25)	2 (0, 7)	25 (6, 74)	9 (2, 28)	743 (696, 907)	477 (357, 544)	6491 (6355, 6629)	3 (1, 10)	3 (1, 9)	36 (26, 37)
**Guinea**	31 (11, 68)	3 (1, 10)	92 (34, 204)	12 (4, 27)	1094 (1024, 1334)	700 (525, 799)	28546 (28410, 28680)	21 (8, 46)	7 (2, 14)	112 (79, 116)
**Guinea-Bissau**	30 (10, 67)	3 (1, 9)	90 (32, 202)	12 (4, 27)	1108 (1037, 1351)	713 (534, 813)	28546 (28410, 28680)	21 (7, 46)	6 (2, 15)	117 (83, 122)
**Guyana**	24 (10, 50)	5 (2, 15)	71 (31, 151)	27 (12, 57)	1016 (951, 1240)	656 (492, 748)	9223 (9086, 9361)	8 (4, 17)	7 (3, 15)	28 (20, 29)
**Haiti**	20 (5, 57)	2 (0, 9)	61 (14, 173)	23 (5, 65)	1018 (952, 1241)	649 (486, 740)	9223 (9086, 9361)	13 (3, 37)	12 (3, 33)	100 (71, 104)
**Honduras**	5 (1, 17)	1 (0, 4)	16 (4, 50)	6 (1, 19)	739 (691, 901)	474 (355, 541)	6491 (6355, 6629)	2 (0, 7)	2 (0, 6)	22 (16, 23)
**India**	18 (8, 35)	4 (1, 10)	53 (24, 104)	20 (9, 39)	1564 (1465, 1908)	1011 (757, 1153)	12649 (12513, 12787)	7 (3, 13)	6 (3, 12)	44 (31, 46)
**Indonesia**	10 (4, 24)	2 (1, 7)	31 (11, 72)	12 (4, 27)	1453 (1361, 1772)	943 (707, 1076)	8150 (8012, 8286)	3 (1, 8)	3 (1, 7)	30 (21, 31)
**Iran, Islamic Republic of**	4 (1, 9)	1 (0, 3)	11 (4, 26)	4 (2, 10)	655 (613, 799)	418 (313, 477)	8139 (8001, 8275)	1 (0, 3)	1 (0, 2)	12 (8, 12)
**Iraq**	6 (2, 13)	1 (0, 3)	19 (7, 38)	7 (3, 14)	858 (803, 1046)	556 (416, 634)	8139 (8001, 8275)	3 (1, 6)	2 (1, 5)	30 (21, 31)
**Jamaica**	4 (1, 10)	1 (0, 3)	12 (4, 29)	4 (2, 11)	567 (530, 692)	362 (271, 413)	9223 (9086, 9361)	1 (0, 2)	1 (0, 2)	5 (4, 5)
**Jordan**	4 (2, 10)	1 (0, 3)	13 (5, 29)	5 (2, 11)	656 (613, 800)	417 (313, 476)	8139 (8001, 8275)	1 (0, 3)	1 (0, 3)	11 (8, 11)
**Kenya**	18 (8, 37)	3 (1, 9)	56 (23, 113)	21 (9, 42)	1050 (983, 1280)	676 (507, 771)	15019 (14881, 15157)	9 (4, 17)	8 (3, 15)	54 (38, 56)
**Kiribati**	16 (6, 41)	4 (1, 12)	47 (18, 125)	18 (7, 47)	730 (683, 890)	467 (350, 533)	21500 (21362, 21637)	6 (2, 15)	5 (2, 13)	72 (51, 75)
**Kosovo**	5 (1, 19)	1 (0, 6)	14 (3, 57)	5 (1, 21)	81 (87, 103)	149 (112, 170)	6576 (6438, 6714)	2 (0, 7)	1 (0, 6)	18 (13, 19)
**Kyrgyzstan**	7 (2, 18)	1 (0, 5)	21 (6, 56)	8 (2, 21)	81 (87, 103)	149 (112, 170)	8032 (7894, 8170)	3 (1, 8)	3 (1, 7)	21 (15, 22)
**Lao People's Democratic Republic**	20 (7, 45)	3 (1, 10)	62 (22, 137)	23 (8, 51)	1614 (1511, 1969)	1043 (782, 1190)	8150 (8012, 8286)	11 (4, 24)	10 (3, 21)	87 (62, 90)
**Lesotho**	35 (16, 69)	6 (2, 15)	105 (48, 207)	14 (6, 28)	1171 (1096, 1428)	754 (565, 860)	12653 (12516, 12791)	18 (8, 35)	6 (3, 11)	121 (86, 126)
**Liberia**	34 (11, 74)	5 (1, 14)	102 (34, 224)	38 (13, 84)	934 (873, 1138)	589 (441, 672)	28546 (28410, 28680)	20 (7, 43)	18 (6, 39)	88 (63, 92)
**Madagascar**	20 (7, 45)	3 (1, 8)	61 (20, 137)	23 (7, 52)	863 (808, 1053)	555 (416, 633)	15019 (14881, 15157)	13 (4, 28)	11 (4, 25)	70 (50, 73)
**Malawi**	40 (17, 80)	8 (3, 20)	121 (52, 241)	46 (20, 91)	986 (924, 1203)	638 (478, 727)	15019 (14881, 15157)	18 (8, 36)	16 (7, 32)	78 (55, 81)

Country	Incidence rates (low, high)							Mortality rates (low, high)		
	Pneumonia (non-severe)	Pneumonia (severe)	NPNM (non-severe)	NPNM (severe)	Meningitis	Meningitis sequelae	Acute otitis media	Pneumonia (severe)	NPNM (severe)	Meningitis
**Mali**	37 (15, 80)	5 (2, 14)	113 (45, 241)	15 (6, 32)	1314 (1229, 1602)	843 (631, 961)	28546 (28410, 28680)	23 (9, 50)	7 (3, 16)	133 (94, 139)
**Marshall Islands**	5 (2, 16)	1 (0, 4)	16 (5, 49)	6 (2, 19)	997 (934, 1217)	646 (484, 737)	21500 (21362, 21637)	2 (1, 8)	2 (1, 7)	38 (27, 40)
**Mauritania**	25 (10, 54)	3 (1, 9)	76 (30, 163)	10 (4, 22)	987 (924, 1204)	637 (477, 727)	28546 (28410, 28680)	16 (6, 34)	5 (2, 11)	110 (78, 115)
**Micronesia, Federated States of**	5 (2, 16)	1 (0, 4)	16 (5, 49)	6 (2, 19)	997 (934, 1217)	646 (484, 737)	21500 (21362, 21637)	2 (1, 8)	2 (1, 7)	38 (27, 40)
**Mongolia**	31 (19, 44)	9 (5, 17)	94 (58, 132)	35 (22, 50)	589 (551, 718)	375 (281, 427)	8032 (7894, 8170)	4 (3, 6)	4 (2, 5)	24 (17, 25)
**Morocco**	8 (3, 19)	2 (1, 6)	25 (9, 58)	9 (3, 22)	859 (805, 1048)	555 (416, 633)	8139 (8001, 8275)	3 (1, 7)	3 (1, 6)	24 (17, 25)
**Mozambique**	27 (10, 61)	3 (1, 9)	82 (29, 184)	11 (4, 24)	1225 (1148, 1495)	796 (597, 908)	15019 (14881, 15157)	19 (7, 42)	6 (2, 13)	96 (68, 100)
**Myanmar**	18 (8, 36)	4 (1, 9)	54 (23, 109)	20 (9, 41)	1279 (1197, 1560)	823 (617, 939)	8150 (8012, 8286)	8 (3, 15)	7 (3, 14)	49 (35, 51)
**Namibia**	15 (7, 30)	3 (1, 8)	46 (20, 91)	17 (8, 34)	1087 (1018, 1326)	703 (527, 802)	12653 (12516, 12791)	7 (3, 13)	6 (3, 12)	61 (44, 64)
**Nepal**	8 (3, 18)	1 (0, 4)	24 (8, 54)	9 (3, 20)	1146 (1074, 1399)	746 (559, 851)	12649 (12513, 12787)	4 (1, 10)	4 (1, 9)	29 (21, 31)
**Nicaragua**	9 (2, 26)	2 (0, 7)	26 (6, 79)	10 (2, 30)	743 (696, 907)	477 (357, 544)	6491 (6355, 6629)	3 (1, 10)	3 (1, 9)	35 (25, 37)
**Niger**	59 (25, 120)	9 (3, 24)	177 (77, 362)	24 (10, 48)	1348 (1261, 1644)	862 (646, 983)	28546 (28410, 28680)	33 (14, 68)	10 (5, 21)	158 (112, 164)
**Nigeria**	25 (9, 55)	3 (1, 7)	74 (26, 166)	10 (3, 22)	1385 (1293, 1688)	860 (645, 981)	28546 (28410, 28680)	17 (6, 39)	5 (2, 12)	145 (103, 151)
**Pakistan**	21 (9, 43)	4 (1, 10)	65 (28, 130)	9 (4, 17)	1300 (1216, 1586)	830 (622, 947)	12649 (12513, 12787)	11 (5, 22)	3 (1, 7)	85 (60, 89)
**Palestine, State of**	4 (2, 5)	1 (1, 2)	11 (7, 16)	4 (3, 6)	655 (613, 799)	418 (313, 477)	8139 (8001, 8275)	1 (0, 1)	0 (0, 1)	6 (4, 6)
**Papua New Guinea**	12 (4, 37)	2 (1, 9)	36 (11, 112)	13 (4, 42)	949 (889, 1158)	614 (460, 701)	21500 (21362, 21637)	5 (2, 17)	5 (1, 15)	78 (56, 82)
**Paraguay**	30 (15, 47)	9 (4, 19)	92 (45, 142)	34 (17, 53)	744 (696, 907)	477 (357, 544)	6171 (6033, 6309)	4 (2, 6)	3 (2, 5)	20 (14, 21)
**Peru**	6 (1, 19)	1 (0, 5)	18 (4, 58)	7 (1, 22)	568 (531, 692)	360 (270, 411)	5986 (5850, 6124)	3 (1, 8)	2 (0, 7)	17 (12, 18)
**Philippines**	9 (3, 23)	2 (0, 6)	28 (9, 70)	11 (3, 26)	1271 (1191, 1551)	832 (623, 948)	8150 (8012, 8286)	4 (1, 9)	3 (1, 8)	32 (23, 34)
**Moldova, Republic of**	4 (2, 13)	1 (0, 5)	12 (5, 40)	5 (2, 15)	162 (173, 207)	298 (223, 340)	6576 (6438, 6714)	1 (0, 3)	1 (0, 3)	9 (7, 10)
**Rwanda**	16 (6, 35)	3 (1, 7)	50 (18, 107)	19 (7, 40)	688 (643, 838)	441 (330, 503)	15019 (14881, 15157)	9 (3, 19)	8 (3, 17)	48 (34, 50)
**Samoa**	16 (7, 28)	5 (2, 11)	48 (21, 84)	18 (8, 32)	687 (642, 838)	435 (326, 496)	21500 (21362, 21637)	2 (1, 4)	2 (1, 3)	10 (7, 10)
**Sao Tome and Principe**	26 (12, 48)	5 (2, 13)	77 (37, 145)	29 (14, 55)	920 (861, 1122)	595 (446, 678)	28546 (28410, 28680)	10 (5, 19)	9 (4, 17)	62 (44, 65)
**Senegal**	16 (5, 37)	2 (1, 7)	50 (16, 111)	19 (6, 42)	1124 (1052, 1371)	721 (540, 823)	28546 (28410, 28680)	10 (3, 22)	9 (3, 19)	62 (44, 64)
**Serbia**	1 (0, 3)	0 (0, 1)	3 (1, 10)	1 (0, 4)	81 (87, 103)	149 (112, 170)	5884 (5746, 6020)	0 (0, 1)	0 (0, 1)	2 (2, 2)
**Sierra Leone**	63 (31, 118)	12 (5, 28)	189 (92, 356)	25 (12, 47)	1069 (1000, 1304)	687 (515, 784)	28546 (28410, 28680)	29 (14, 55)	9 (5, 17)	161 (114, 168)
**Solomon Islands**	7 (2, 31)	2 (0, 9)	22 (6, 93)	8 (2, 35)	830 (777, 1012)	533 (400, 608)	21500 (21362, 21637)	2 (1, 10)	2 (1, 9)	30 (21, 31)
**Somalia**	58 (19, 136)	4 (1, 12)	175 (56, 410)	23 (7, 54)	2078 (1944, 2534)	1336 (1001, 1523)	15019 (14881, 15157)	47 (15, 110)	15 (5, 35)	232 (164, 242)
**South Africa**	13 (6, 25)	3 (1, 7)	39 (18, 76)	15 (7, 29)	1042 (975, 1271)	672 (503, 766)	12653 (12516, 12791)	5 (2, 10)	5 (2, 9)	53 (37, 55)
**South Sudan**	32 (13, 68)	5 (2, 12)	98 (41, 205)	13 (5, 27)	848 (793, 1034)	540 (404, 616)	15019 (14881, 15157)	19 (8, 40)	6 (3, 13)	119 (84, 124)

Country	Incidence rates (low, high)							Mortality rates (low, high)		
	Pneumonia (non-severe)	Pneumonia (severe)	NPNM (non-severe)	NPNM (severe)	Meningitis	Meningitis sequelae	Acute otitis media	Pneumonia (severe)	NPNM (severe)	Meningitis
**Sri Lanka**	2 (1, 5)	0 (0, 1)	6 (2, 15)	2 (1, 6)	851 (798, 1039)	556 (417, 634)	8150 (8012, 8286)	1 (0, 2)	1 (0, 2)	4 (3, 4)
**Sudan**	25 (8, 54)	4 (1, 10)	74 (25, 164)	28 (9, 62)	859 (804, 1047)	555 (416, 633)	15019 (14881, 15157)	14 (5, 32)	13 (4, 28)	100 (71, 105)
**Swaziland**	22 (9, 45)	4 (1, 10)	66 (27, 134)	25 (10, 51)	791 (740, 964)	504 (378, 575)	12653 (12516, 12791)	10 (4, 21)	9 (4, 19)	87 (62, 90)
**Syrian Arab Republic**	4 (2, 5)	1 (1, 2)	11 (7, 16)	4 (3, 6)	655 (613, 799)	418 (313, 477)	8139 (8001, 8275)	1 (0, 1)	0 (0, 1)	6 (4, 6)
**Tajikistan**	19 (7, 41)	3 (1, 10)	59 (22, 125)	22 (8, 47)	81 (87, 103)	149 (112, 170)	8032 (7894, 8170)	9 (4, 20)	8 (3, 18)	61 (43, 63)
**Macedonia, the former Yugoslav Republic of**	6 (3, 13)	2 (1, 5)	17 (8, 39)	6 (3, 15)	81 (87, 103)	149 (112, 170)	5884 (5746, 6020)	1 (0, 2)	1 (0, 2)	3 (2, 3)
**Thailand**	3 (1, 6)	1 (0, 2)	8 (4, 18)	3 (1, 7)	750 (701, 915)	478 (358, 545)	8150 (8012, 8286)	1 (0, 2)	1 (0, 1)	6 (4, 6)
**Timor-Leste**	23 (10, 47)	5 (2, 12)	71 (30, 141)	27 (11, 53)	1206 (1127, 1471)	761 (570, 868)	8150 (8012, 8286)	10 (4, 19)	9 (4, 17)	83 (59, 86)
**Togo**	30 (12, 64)	4 (1, 11)	91 (37, 192)	12 (5, 26)	1093 (1023, 1334)	703 (527, 802)	28546 (28410, 28680)	18 (7, 39)	6 (2, 12)	95 (68, 99)
**Tonga**	24 (11, 42)	7 (3, 16)	71 (32, 126)	27 (12, 47)	670 (626, 817)	425 (319, 485)	21500 (21362, 21637)	3 (1, 5)	3 (1, 5)	13 (9, 14)
**Tunisia**	2 (0, 4)	0 (0, 1)	5 (1, 13)	2 (1, 5)	655 (613, 799)	418 (313, 477)	8139 (8001, 8275)	1 (0, 2)	1 (0, 2)	7 (5, 8)
**Turkmenistan**	12 (4, 27)	2 (1, 6)	38 (14, 82)	14 (5, 31)	81 (87, 103)	149 (112, 170)	8032 (7894, 8170)	6 (2, 14)	6 (2, 12)	67 (47, 70)
**Tuvalu**	24 (11, 42)	7 (3, 16)	71 (32, 126)	27 (12, 47)	670 (626, 817)	425 (319, 485)	21500 (21362, 21637)	3 (1, 5)	3 (1, 5)	13 (9, 14)
**Uganda**	39 (20, 71)	9 (4, 21)	119 (61, 216)	45 (23, 81)	1036 (970, 1264)	667 (500, 761)	15019 (14881, 15157)	14 (7, 26)	13 (6, 23)	71 (50, 74)
**Ukraine**	3 (1, 7)	1 (0, 3)	8 (3, 21)	3 (1, 8)	162 (173, 207)	298 (223, 340)	6576 (6438, 6714)	0 (0, 1)	0 (0, 1)	4 (2, 4)
**Tanzania, United Republic of**	30 (14, 57)	6 (2, 15)	90 (42, 173)	34 (16, 65)	1138 (1066, 1389)	737 (552, 841)	15019 (14881, 15157)	12 (6, 24)	11 (5, 21)	66 (46, 68)
**Uzbekistan**	13 (5, 25)	3 (1, 7)	38 (16, 77)	14 (6, 29)	81 (87, 103)	149 (112, 170)	8032 (7894, 8170)	5 (2, 11)	5 (2, 10)	42 (30, 43)
**Vanuatu**	7 (2, 29)	2 (0, 9)	21 (5, 88)	8 (2, 33)	1040 (973, 1269)	667 (500, 761)	21500 (21362, 21637)	2 (1, 10)	2 (1, 9)	25 (18, 26)
**Venezuela, Bolivarian Republic of**	5 (1, 15)	1 (0, 5)	16 (4, 45)	6 (2, 17)	566 (530, 691)	362 (271, 413)	6491 (6355, 6629)	2 (0, 5)	1 (0, 4)	9 (6, 10)
**Viet Nam**	12 (5, 27)	3 (1, 9)	37 (16, 80)	14 (6, 30)	1082 (1012, 1319)	689 (516, 786)	8150 (8012, 8286)	3 (1, 7)	3 (1, 6)	19 (13, 20)
**Yemen**	9 (2, 21)	1 (0, 3)	26 (7, 63)	10 (3, 24)	860 (805, 1049)	555 (416, 633)	8139 (8001, 8275)	6 (2, 14)	5 (1, 13)	47 (33, 49)
**Zambia**	29 (13, 58)	6 (2, 15)	89 (39, 175)	33 (15, 66)	1298 (1215, 1583)	834 (625, 951)	15019 (14881, 15157)	13 (6, 25)	11 (5, 22)	87 (62, 91)
**Zimbabwe**	33 (13, 69)	5 (2, 15)	98 (38, 207)	37 (14, 78)	1081 (1012, 1319)	697 (522, 795)	12653 (12516, 12791)	17 (7, 36)	15 (6, 32)	95 (67, 99)

2.3 DALYs weights for each disease

For each birth cohort under five, disability-adjusted life years (DALYs) were estimated in this model by taking the sum of the number of years lived with disability and years of life lost. The number of years lived in disability was estimated by multiplying the number of cases by the DALYs weights, the percentage of healthy time lost, from GBD 2019 [40] and the duration of illness (assumed as with the UNIVAC model). The years of life lost was estimated by multiplying the number of deaths by the life expectancies taken from the VIMC secretariat, obtained from the UN World Population Prospects.[41]

2.4 Probabilistic analysis

Table 4. Parameters for probabilistic sensitivity analysis

Parmeter	Base case	Probability distribution	Source
**Disease burden estimates (<5 years)**			
Incidence rate			
Sp pneumonia (non-severe)<br>Sp pneumonia (severe)<br>Sp NPNM (non-severe)<br>Sp NPNM (severe)<br>Sp Meningitis	Country-specific	Beta-PERT (mid = country-specific value, range = reported uncertainty range)	4
Sp Meningitis sequelae			32
Sp AOM	Region-specific	Beta-PERT (mid = region-specific value, range = reported uncertainty range)	33
Mortality rate			
Sp pneumonia (severe)<br>Sp NPNM (severe)<br>Sp Meningitis	Country-specific	Beta-PERT (mid = country-specific value, range = reported uncertainty range)	4
**Disability weights for DALYS**			
Percentage of healthy time lost when diseased			
Sp pneumonia (non-severe)	0.051 (0.032, 0.074)	Beta-PERT(mid = GBD 2019, moderate lower respiratory infection, range = GBD 2019 95% uncertainty interval)	40
Sp pneumonia (severe)	0.133 (0.088, 0.19)	Beta-PERT(mid = GBD 2019, severe lower respiratory infection, range = GBD 2019 95% uncertainty interval)	
Sp NPNM (non-severe)	0.051 (0.032, 0.074)	Beta-PERT(mid = GBD 2019, moderate lower respiratory infection, range = GBD 2019 95% uncertainty interval)	
Sp NPNM (severe)	0.133 (0.088, 0.19)	Beta-PERT(mid = GBD 2019, meningitis, range = GBD 2019 95% uncertainty interval)	
Sp Meningitis	0.133 (0.088, 0.19)	Beta-PERT(mid = GBD 2019, meningitis, range = GBD 2019 95% uncertainty interval)	
Sp Meningitis sequelae	0.26 (0.153, 0.364)	Beta-PERT(mid = assumption, range = assumption)	
Sp AOM	0.013 (0.007, 0.024)	Beta-PERT(mid = GBD 2019, acute otitis media, range = GBD 2019 95% uncertainty interval)	
Average duration of illness			

Parmeter	Base case	Probability distribution	Source
Sp pneumonia (non-severe)	7 days (7, 7)	Beta-PERT(mid = assumption, range = assumption)	
Sp pneumonia (severe)	10 days (7, 21.1)	Beta-PERT(mid = assumption, range = assumption)	
Sp NPNM (non-severe)	7 days (7, 7)	Beta-PERT(mid = assumption, range = assumption)	
Sp NPNM (severe)	10 days (7, 21.1)	Beta-PERT(mid = assumption, range = assumption)	Assumptions
Sp Meningitis	10 days (7, 21.1)	Beta-PERT(mid = assumption, range = assumption)	
Sp Meningitis sequelae	50 years (50, 50)	Beta-PERT(mid = assumption, range = assumption)	
Sp AOM	7 days (7, 7)	Beta-PERT(mid = assumption, range = assumption)	
**Vaccine efficacy for Sp AOM**			
Dose 1	0.1 (0.035, 0.19)	Beta-PERT(mid = half effectiveness of Eskola trial for PCV7, range = 95% uncertainty interval)	[21]
Dose 2 and Dose 3	0.2 (0.07, 0.31)	Beta-PERT(mid = Eskola trial for PCV7, range = 95% uncertainty interval)	
**Incidence rate ratio (IRR)**			
North America	0.27 (0.22, 0.33)		
Latin America	0.50 (0.43, 0.58)		
Africa	0.67 (0.47, 0.86)	1000 posterior samples for each region	[10]
Europe	0.50 (0.42, 0.59)		
Asia	0.66 (0.47, 0.87)		
Oceania	0.55 (0.27, 0.85)		

Sp: Streptococcus pneumoniae

2.5 Healthcare cost

The price per dose was assumed to be $15·68 for non-Gavi eligible countries and $3·05 (plus 20% tail price) for Gavi-eligible countries. A freight cost of 6% of the vaccine price, a 5% wastage rate, and a buffer stock of 25% of the first-year vaccine needs were included. Vaccine administration using five minutes of nurses' time to administer the vaccine and related injection supplies were included. Health-care costs included treatment in the hospital for all disease presentations except acute otitis media, for which outpatient costs were used. We also accounted for care-seeking behaviour for nIPD pneumonia and acute otitis media. We used the cost estimates as our earlier paper for comparability,[1] where costs for meningitis in low-income and middle-income countries were extracted.[42] Costs for pneumonia were modelled from data in the WHO-CHOICE database,[43] and a 2016 systematic review.[44] All unit prices were converted to 2015 international dollars ($), to facilitate comparisons with our previous findings.[1]

The costs of treating a patient with pneumococcal disease (meningitis, pneumonia, or NPNM invasive pneumococcal disease) were predicted using the best-fit model generated from data extracted from the literature. PCV cost-effectiveness analysis (CEA) studies were found through literature review and/or included in four main systematic reviews; from those two costing studies and 27 CEA studies were selected and used.[42,44-75] The costs were extracted and converted into International dollars at corresponding years the studies reported and then adjusted to 2015 using the ratio between GDP per capita PPP in year 2015 and those corresponding years.

We used international dollar I$, as it is widely used for international comparisons, and we present results mainly on a regional basis. The I$ was comparable to the amount of goods and services a U.S. dollar would buy in the United States for the cited country/Region, accounting for its purchasing power. Other relevant similar studies have also used I$, for example, Sinha's study on 72 developing countries: Sinha A, Levine O, Knoll MD, Muhib F, Lieu TA. Cost-effectiveness of pneumococcal conjugate vaccination in the prevention of child mortality: an international economic analysis. Lancet 2007; 369: 389–96.

Meningitis
Hospitalisation costs for Meningitis in low-middle income countries (LMIC) were extracted from a study by Portnoy et al that predicted costs for all LMICs by conducting a systematic review and extrapolating data to set up a database on cost of care for childhood meningitis.[42]

Pneumonia
Hospitalisation costs for pneumonia were extracted from a study by Zhang et al., which conducted a systematic review of studies providing the cost of childhood pneumonia and also included unpublished data at that time.[44] In their study the cost for LMICs was extracted from severe pneumonia data which mean is 242·7 USD 2013 (and also I$ 2013). As costs per episode for each country, were not available, we used WHO-CHOICE inpatient costs per day costs per day multiplied by 3·9 days as a proxy for costs per episode.[76] The 3·9 days value was the average length of stay for pneumonia disease based on average length of stay for pneumonia in the United States.[77] The resulting values were converted to International dollars of year 2015 (I$ 2015). To incorporate this information, we used weighted averages, with weights being the population size under five years of age, and the resulting weighted costs were compared with the extracted costs from Zhang et al.[44] We use the resulting value as a multiplicative factor. Finally, we applied this factor to the costs that we have generated. The multiplicative factor for LMIC was 1·18.

NPNM

To estimate hospitalisation costs for NPNM, costs from 25 PCV CEA studies were extracted (N = 29 countries).[48-56,58-65,67,69-75] As the available costs were skewed, we used the logarithm of costs as our outcome. The predictors used were the logarithm of GDP per capita PPP in year 2015 and UN regions indicators. In search for the best fitting model, we used stepwise regression with AIC criterion. The resulting model was:

$$\ln(\text{cost}) = 4{\cdot}33 + 0{\cdot}41*\ln(\text{GDP per capita PPP}) – 0{\cdot}73*I(\text{Asia} = 1) – 2{\cdot}08*I(\text{Africa} = 1)$$

The adjusted R-squared was 65·5%. To transform back to cost, we needed to have a correcting factor.[78] The correcting factor equals to the sum of the expected residuals. In this case, it was 1·108. Therefore, the cost model used was:

$$\text{Cost} = \exp(4{\cdot}33 + 0{\cdot}41*\ln(\text{GDP per capita PPP}) – 0{\cdot}73*I(\text{Asia} = 1) – 2{\cdot}08*I(\text{Africa} = 1))*1{\cdot}108$$

Through this model, we predicted the costs of all the countries, including the 29 countries whose data were used to generate the model.

AOM

Outpatient treatment costs for AOM were extracted from 24 PCV CEA studies (N = 25 countries).[47-49,51-56,58-60,62-67,69-71,73-75] As the resulting costs were skewed, we used the logarithm of costs as our outcome. The predictors used were the logarithm of GDP per capita PPP in year 2015 and UN regions indicators. In search for the best fitting model, we use stepwise regression with AIC criterion. The resulting model was:

$$\ln(\text{cost}) = -5{\cdot}46 + 0{\cdot}94*\ln(\text{GDP per capita PPP}) + 0{\cdot}45*I(\text{Asia} = 1) – 0{\cdot}89*I(\text{Oceania} = 1)$$

The adjusted R-squared was 42·8%. To transform back to cost, we had a correcting factor.[78] The correcting factor equals to the sum of the expected residuals. Here, it was 1·218. Therefore, the cost model was:

$$\text{Cost} = \exp(-5{\cdot}46 + 0{\cdot}94*\ln(\text{GDP per capita PPP}) + 0{\cdot}45*I(\text{Asia} = 1) – 0{\cdot}89*I(\text{Oceania} = 1))*1{\cdot}218$$

Through this model, we predicted the costs of all the countries, including the 25 countries whose data were used to generate the model.

Table 5 below summarises the average healthcare cost across diseases and regions.

Table 5. Mean healthcare cost by disease and region

	Pneumonia	NPNM	Meningitis	Acute otitis media
Global	207	1290	2280	32.4
	(10.1-740)	(146-5000)	(122-12600)	(2.16-83.6)
Africa	106	303	1140	13.8
	(10.1-522)	(146-544)	(122-8890)	(2.16-44.5)
Asia	250	1640	2680	42.5
	(32.6-566)	(912-2270)	(217-9150)	(10.3-84.6)
Europe	390	3680	5860	31.3
	(146-741)	(2800-4690)	(2580-11600)	(16.2-53.0)
Latin America	394	3780	5450	34.2
	(38.7-589)	(1820-5000)	(170 – 12600)	(6.01-61.4)
Oceania	143	2460	961	5.09
	(92.7-221)	(1860-3580)	(295-5070)	(2.59-11.7)

2.6 Incremental cost-effectiveness ratios (ICERs)

We estimated the incremental cost-effectiveness ratio (ICER) by comparing the introduction of PCV-13 to no PCV use or [(Cost in PCV-13 – Cost in no vaccination)/(DALYs averted)]. The mean of 1000 ICERs bootstraps were calculated on International dollars I$ (2015) and DALYs. We compared with different willingness-to-pay thresholds. However, as the willingness-to-pay study was US-based, it might not be representative of low- and middle-income countries. As such, we considered using a previously common rule of thumb, where an intervention is cost-effective if a healthy year is gained at less than three times the GDP per capita.[79] We also compared the resulting ICERs with the GDP per capita (PPP) as it has been traditionally used as an indicative threshold to indicate when an intervention is cost-effective.[80] The ICERs were compared with the GDP per capita (PPP) of each region, calculated by summing over population-weighted figures for each country. **Table 6** presents the undiscounted rates. The global ICER is estimated to be $821 per DALYs averted. If PCV coverage is increased to DPT coverage levels from 2020, it will require more investment in vaccination especially for countries without PCV, and the ICER will increase to $1310 per DALY averted. This is due to the increased cost of vaccination, particularly for countries without PCV to be protected for the next 10 years to 2030. However, in the full protection scenario, the ICER will drop to $657 per DALY averted due to the huge reduction in DALYs across 30 years of protection. All the resulting ICERs are presented with a 3% discount rate applied to costs and utility outcomes in the manuscript.

Table 6. Incremental cost-effectiveness ratios compared to no vaccination scenario ($ per DALYs averted, undiscounted) over 30 years, from the health-system perspective (undiscounted).

	**PCV coverage**	**DPT coverage**	**Full protection**
**DALYs averted (millions)**			
Global	46.0 (24.0-68.9)	56.5 (30.9-83.3)	264 (210-312)
Africa	33.5 (12.9-54.7)	36.0 (13.8-58.7)	141 (112-167)
Asia	10.7 (4.18-17.6)	18.7 (7.29-30.9)	114 (91.1 – 135)
Europe	0.0232 (0.0152-0.0334)	0.0365 (0.0236-0.0538)	0.259 (0.184-0.343)
Latin America	1.61 (1.12-2.15)	1.71 (1.18-2.29)	7.02 (5.32-8.70)
Oceania	0.105 (0.0345-0.178)	0.108 (0.0354-0.182)	0.923 (0.735-1.11)
**Healthcare cost saved ($ billions)**			
Global	3.19 (2.19-4.3)	6.48 (4.27-8.81)	23.1 (19.5-27.2)
Africa	1.39 (0.789-1.98)	1.53 (0.869-2.17)	3.76 (3.19-4.41)
Asia	1.23 (0.66-1.83)	4.29 (2.37-6.22)	17.5 (14.9-20.4)
Europe	0.00547 (0.00318-0.00886)	0.0274 (0.02-0.0366)	0.141 (0.112-0.176)
Latin America	0.547 (0.373-0.752)	0.609 (0.418-0.836)	1.59 (1.17-2.09)
Oceania	0.0154 (0.00608-0.0275)	0.0163 (0.00643-0.0289)	0.0687 (0.0459-0.0977)
**Vaccination cost ($ billions)**			
Global	38.0	75.4	194
Africa	19.9	24.9	52.2
Asia	13.4	44.9	130
Europe	0.113	0.588	1.89
Latin America	4.46	5.04	10.0
Oceania	0.0602	0.0699	0.200
**Incremental cost-effectiveness ratios ($ per DALY adverted)**			
Global	$821 ($492-$1480)	$1310 ($801-$2290)	$657 ($535-$833)
Africa	$620 ($328-$1470)	$726 ($386-$1720)	$346 ($286-$436)
Asia	$1370 ($655-$3030)	$2620 ($1250-$5800)	$997 ($813-$1260)
Europe	$4840 ($3120-$7180)	$16100 ($10300-$24100)	$6940 ($5000-$9640)
Latin America	$2510 ($1730-$3650)	$2680 ($1840-$3900)	$1220 ($911-$1660)
Oceania	$507 ($184-$1500)	$588 ($224-$1720)	$146 ($92.4-$210)

3. COMPARISONS WITH PREVIOUS MODELS

Table 7 summarises the key parameters between full protection model (2019) and the current model, including key parameters:

Table 7. Parameters from the full protection model in 2019 and our current model

	Full protection (2019)	Current model
**PCV Coverage**	Countries assumed to have full PCV coverage	Countries' actual PCV coverage
**Vaccine impact**	Vaccine efficacy differs by the six United Nations (UN) regions (Africa, Asia, Europe, Latin American and the Caribbean, North America and Oceania), where we assumed that countries in the same region would have the same incidence risk ratios	Differs by country as it depends on PCV coverage, vaccine timeliness, vaccine efficacy
**Time take to near elimination of VT IPD**	Two years	4·6 years (95% CrI: 3·9-6·0)
**Vaccine coverage required to reach full vaccine impact**	Full impact from 2nd year onwards	Requires at least 82% PCV coverage to reach full vaccine impact
**PCV impact on nIPD (pneumonia)**	Same as IPD	60% of the impact on IPD
**PCV impact on nIPD (AOM)**	19%	20%

We compared the results using five different scenarios (**Figure 4**). First, the updated pseudo-dynamic algorithm with vaccine coverage was used. The updated pseudo-dynamic algorithm is the main method used in the study and combines the two previous models by applying the Flasche algorithm [10] with the UNIVAC model. Second, we incorporated real-world PCV coverage and year of vaccination introduction into the previous pseudo-dynamic algorithm from Chen et al. [1] where the regional incidence risk ratios (IRRs) were applied using Flasche algorithm [10]. The model simplifies long-term impact predictions, encompassing serotype replacement and herd protection, by condensing them into a single predictive equation. The original model involves certain assumptions: (a) the use of PCV at a specific coverage level, though not necessarily reaching 100% coverage (which illustrates our the herd immunity dynamics), (b) the eliminated serotypes will be entirely replaced in carriage by non-vaccine serotypes, and (c) the propensity of the non-vaccine serotype group to cause invasive disease, if carried, remains constant in the post-PCV era.

The resulting predicted incidence rate ratio are as follows:

$$IRR = \frac{\lambda c + 1}{d + 1}$$

c is the odds of vaccine-type carriage
d is the odds of IPD
λ is the proportion of vaccine-type carriage replaced by non-vaccine-type (our study assumed to be 1)

Thus, under these assumptions, the predicted IRR can be treated as the maximum reduction in IPD achievable from a vaccination programme. The pseudo-dynamic model was validated using serotype-specific carriage in LMICs[81] and post-vaccine introduction data from high-income countries, which we extrapolated to LMICs due to a lack of post-introduction data from these settings. Since then, new LMIC data have emerged, allowing us to refine our assumptions.

For the vaccine coverage, we used real-world PCV coverage from 2000 to 2019 and 2019 PCV coverage for future years. Third, the UNIVAC model, a commonly used model, was used [2,3]. Fourth, using the updated pseudo-dynamic algorithm, we used under-1 DTP vaccine coverage [6] from 2000 to 2019 and 2019 DTP coverage for future years. Lastly, we considered the previous pseudo-dynamic algorithm from Chen et al.[1] with full protection. The percentage reductions are presented in **Table 8**.

The PCV impact on nIPD pneumonia was larger in the 2019 model as the 2019 model assumed the same vaccine impact for IPD and nIPD (**Figure 4**). The other two improvements to the updated model, namely (i) the near elimination of VT IPD and (ii) vaccine coverage required to reach full vaccine impact, while useful to incorporate real-world evidence, did not have an influential impact on IPD burden (**Figure 4** (solid and dotted red line); **Table 7**).

Our updated model also accounted for the longer time needed to achieve maximum impact since PCV introduction. Furthermore, the year of PCV introduction based on actual data was assumed to start much later (**Table 2**) than 2000 in our previous model. We also assumed declining mortality rates over time due to improved living standards.[2] When we updated our previous model to include the year of PCV introduction and PCV coverage, the relative impact of PCV was similar to current findings (**Table 8** and **Figure 4**). Our analysis also estimates smaller relative reductions in pneumococcal burden compared to previous estimates from the Vaccine Impact Modelling Consortium (VIMC),[82] where the authors assume continued improvements in PCV coverage and new country introductions to 2030. Thus, the delays in vaccine introduction and lower PCV coverage rates in LMICs have cost the lives of many children under-5.

Our analysis also estimates smaller reductions in pneumococcal burden compared to previous estimates from the Vaccine Impact Modelling Consortium (VIMC),[82,83] which estimated that PCV would avert 2.8 million deaths and 190 million DALYs in 112 highest-burden countries in 2000-2030. These rely on two static models (UNIVAC and the Lives Saved Tool), neither of which incorporate indirect effects like herd immunity and serotype replacement. While herd immunity would be expected to increase vaccine impact, most of the effect is in older adults which are not captured in any of the analyses and only constitute a small portion of the pneumococcal burden in LMICs. In addition, a recent systematic review conducted amongst under-5 in low- and middle-income

countries found that although PCV introduction decreased the prevalence of vaccine-type (VT) carriage, the prevalence of non-vaccine-type (NVT) carriage has been shown to increase due to serotype replacement, where carriage of NVTs could increase up to 74.1% post-vaccination [7]. As such, serotype replacement would diminish vaccine impact. Additionally, the VIMC models assumed continued improvements in PCV coverage and new country introductions to 2030, whereas we assumed that coverage would remain static after 2019. Research in LMICs also reports a limited number of cases involving vaccine serotypes continue to be transmitted. In South Africa, the incidence rate of PCV7 serotype IPD among children under-2 decreased from 32.1 to 3.4 cases per 100,000 person-years when PCV7 coverage reached 81%.[84] This trend was mirrored in Kenya, where the PCV10-serotype IPD incidence rate dropped from 60.8 to 3.2 cases per 100,000 person-years with PCV10 coverage at 87%.[16] Other studies have also projected similar outcomes regarding the persistence of vaccine types. In Mongolia, despite achieving 100% vaccine coverage, there was a decrease in vaccine-type carriage from 29.1% to 13.1%.[19] In Nigeria, a prevalence drop from 21% to 12% was observed with 84% vaccine coverage.[85]

There are other limitations in addition to those mentioned in the main paper. Firstly, the carriage in under-5s could be influenced by post-vaccination changes in the immune status of people older than five years, which we did not directly account for. Indeed, a study in Kilifi, Kenya, estimated that the main contributor to the force of pneumococcal infection was school-aged children [86]. By ignoring these second-tier effects (under-5 vaccination reducing carriage in older children, which further reduces carriage in under-5s), our estimates might be more conservative. Next, since quantitative evidence on herd protection and serotype (and species) replacement for AOM are sparse, we modelled vaccine impact on AOM using a fixed vaccine efficacy of 20% instead of trying to model serotype dynamics. As such, unlike the other disease outcomes, we could only account for the direct impact of vaccination on AOM. As vaccine coverage can also vary significantly across different settings, by assuming uniform coverage, we may not have accounted for the disparities in vaccine access within countries. Notably, many countries that have yet to introduce PCV into their routine schedules are middle-income countries with no access to pooled procurement, possibly indicating that high vaccine prices may be a barrier to wider introduction. We encourage future research to explore these nuances in greater detail for countries where data is available to provide a more comprehensive understanding of the impact of pneumococcal vaccination within the country. In addition, previously, thresholds of 1 to 3 times GDP per capita were commonly used in global analyses, but they have now been widely criticized for failing to represent actual health opportunity costs in most countries.[79,87]

Figure 4. Comparison of outcomes for 112 countries by diseases IPD and nIPD pneumonia across different impact methods.

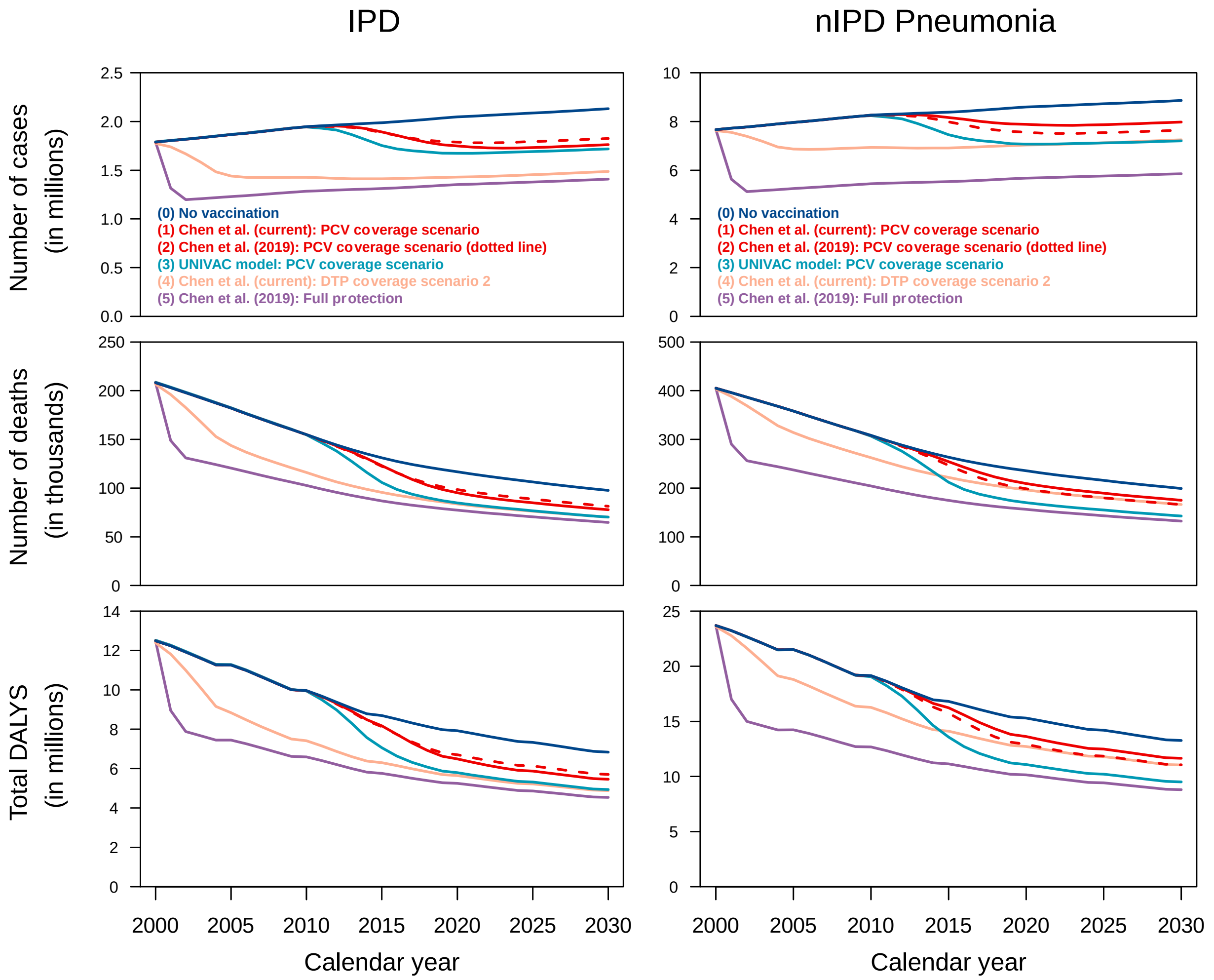

IPD: invasive pneumococcal diseases, which represents the sum of pneumococcal meningitis, pneumococcal non-pneumonia, non-meningitis, and invasive pneumococcal pneumonia

nIPD: non-invasive pneumococcal diseases

PCV: pneumococcal conjugate vaccine

(0) **No vaccination**.

(1) Chen et al. (current)**: PCV coverage scenario**: Using the updated pseudo-dynamic algorithm, respective year's PCV coverage data from 2000 to 2019, and 2019 PCV coverage data from 2020 to 2030.

(2) Chen et al. (2019)**: PCV coverage scenario (dotted line)**: Using the previous pseudo-dynamic algorithm, respective year's PCV coverage data from 2000 to 2019, and 2019 PCV coverage data from 2020 to 2030. Vaccine efficacy was assumed to the same for IPD and nIPD Pneumonia.

(3) **UNIVAC model: PCV coverage scenario**: UNIVAC (adjusted for vaccine coverage and vaccine efficacy), exclude indirect effects from García et al. [2,3]). Using respective year's PCV coverage data from 2000 to 2019, and 2019 PCV coverage data from 2020 to 2030.

(4) Chen et al. (current)**: DTP coverage scenario (2)**: Using the updated pseudo-dynamic algorithm, respective year's DTP coverage data from 2000 to 2019, and 2019 DTP coverage data from 2020 to 2030.

(5) Chen et al. (2019)**: Full protection**: Regional incidence risk ratios (IRRs) applied using Flasche algorithm [10]. This model assumes full protection with the elimination of vaccine serotypes and herd protection.

Table 8. Percentage of cases, deaths, and DALYs averted (mean, 95% CrI) vaccine coverage (2000 to 2030)

S/N	Model	Coverage	Assumptions about indirect effects		Percentage averted (%) (compared with no vaccination)		
				**Disease**	**Cases**	**Deaths**	**DALYs**
(1)	Chen et al. (current)	Real-world PCV coverage for 2000-2019, then constant coverage	Herd immunity and serotype replacement, without complete VT IPD elimination	**IPD**	7.90	7.23	7.53
				**nIPD Pneumonia**	4.48	4.39	4.56
(2)	Chen et al. (2019)	Real-world PCV coverage for 2000-2019, then constant coverage	Herd immunity and serotype replacement, without complete VT IPD elimination	**IPD**	6.82	6.25	6.52
				**nIPD Pneumonia**	6.47	6.33	6.58
(3)	UNIVAC	Real-world PCV coverage for 2000-2019, then constant coverage	No herd immunity or serotype replacement	**IPD**	10.24	11.92	12.34
				**nIPD Pneumonia**	9.74	12.11	12.53
(4)	Chen et al. (current)	Real-world DTP coverage for 2000-2019, then constant coverage	Herd immunity and serotype replacement, without complete VT IPD elimination	**IPD**	25.79	22.27	23.00
				**nIPD Pneumonia**	15.52	13.13	13.52
(5)	Chen et al. (2019)	100% from 2000 (Full protection)	Herd immunity and serotype replacement, with complete VT IPD elimination	**IPD**	32.80	31.78	32.00
				**nIPD Pneumonia**	32.81	31.80	31.99

IPD: invasive pneumococcal diseases, which represents the sum of pneumococcal meningitis, pneumococcal non-pneumonia, non-meningitis, and invasive pneumococcal pneumonia
nIPD: non-invasive pneumococcal diseases
DTP: diphtheria-tetanus-pertussis
PCV: pneumococcal conjugate vaccine
(1) and (4) Chen et al. (current): Combined model using Flasche algorithm [10] and vaccine timeliness from the UNIVAC model from García et al [2,3]). Assumptions: (i) Herd immunity and serotype replacement, (ii) complete VT IPD elimination, (iii) 82.1% vaccine coverage required to reach full vaccine impact and (iv) differentiated PCV impact on nIPD pneumonia. Details were explained in the methods section.
(2) and (5) Chen et al. (2019): previous model using regional incidence risk ratios (IRRs) from Flasche algorithm [10]. Assumptions: (i) Herd immunity and serotype replacement, (ii) without complete VT IPD elimination, (iii) 100% vaccine coverage required to reach full vaccine impact and (iv) same PCV impact on IPD and nIPD pneumonia.
(3) UNIVAC model: UNIVAC (adjusted for vaccine coverage and vaccine efficacy), exclude indirect effects from García et al [2,3]). Vaccine coverage and pneumococcal disease age distributions were estimated by week of age <5 years (rather than averaged for each full year of age). The model assumed 58% vaccine efficacy against all types of IPD using estimates from a meta-analysis of clinical trials. The model did not adjust for indirect effects (herd effects, type replacement) over time.

Table 9 presents the outcomes (cases, deaths and DALYs) in the no vaccination and vaccination scenarios. From 2000 to 2030, we projected a total of 2 570 (95% credible interval (CrI): 2 530-2 610) million cases, 13·1 (95% CrI: 10·4-15·4) million deaths, and 824 (95% CrI: 656-975) million DALYs in the no vaccination scenario. With the introduction of PCV using countries' specific vaccine coverage data, we projected 2 430 (95% CrI: 2 380-2 490) million cases, 12.4 (95% CrI: 9·90-14·6) million deaths, and 778 (95% CrI: 621-923) million DALYs. If the vaccine coverage levels between 2020 to 2030 were increased to 2019 DTP levels, we estimated an additional 51·7 (95% CrI: 34·7-68·2) million cases, 146 (95% CrI: 75·5-219) thousand deaths and 10·5 (95% CrI: 5·38-16·1) million DALYs to be averted globally. Acute otitis media had the largest number of additional cases averted at 43·5 (95% CrI: 27·5-59·2) million cases, while nIPD pneumonia had the largest number of additional deaths and DALYs averted at 77·6 (95% CrI: 42·1-113) thousand and 5·41 (95% CrI: 2·85-7·96) million respectively.

Table 9. Cases, deaths, DALYs, and healthcare costs by regions and diseases breakdown in no vaccination and PCV introduction scenario (undiscounted).

	(1) No vaccination scenario (Mean, 95% CrI)				(2) PCV introduction at PCV coverage (Mean, 95% CrI)				(3) PCV introduction at DTP coverage from 2020 (Mean, 95% CrI)			
**Region**	**Cases (in millions)**	**Deaths (in millions)**	**DALYs (in millions)**	**Healthcare cost ($ bil)**	**Cases (in millions)**	**Deaths (in millions)**	**DALYs (in millions)**	**Healthcare cost ($ bil)**	**Cases (in millions)**	**Deaths (in millions)**	**DALYs (in millions)**	**Healthcare cost ($ bil)**
**Global**	2 570 (2 530-2 610)	13.1 (10.4-15.4)	824 (656-975)	86.2 (75.6-98.3)	2 430 (2 380-2 490)	12.4 (9.9-14.6)	778 (621-923)	83 (73.1-94.6)	2 380 (2 320-2 450)	12.2 (9.81-14.4)	768 (614-911)	79.7 (70-91.1)
**IPD**	61.4 (50-74.5)	4.36 (2.89-6)	282 (185-389)	24.9 (17-33.9)	56.6 (46.1-68.9)	4.05 (2.69-5.59)	261 (172-363)	23.4 (16.1-31.9)	54.3 (44.2-66.4)	3.98 (2.65-5.51)	256 (169-357)	22.2 (15.2-30.4)
**nIPD Pneumonia**	259 (234-287)	8.7 (7.55-9.39)	542 (470-585)	28.8 (26-31.9)	248 (224-275)	8.32 (7.22-9.01)	517 (449-560)	28 (25.4-31)	242 (218-269)	8.24 (7.15-8.93)	512 (444-555)	27 (24.4-30.1)
**Acute otitis media**	2 240 (2 240-2 240)	0 (0-0)	0.56 (0.56-0.56)	32.6 (32.6-32.6)	2 130 (2 090-2 170)	0 (0-0)	0.531 (0.521-0.541)	31.6 (31.3-32)	2 090 (2 030-2 140)	0 (0-0)	0.52 (0.506-0.534)	30.5 (29.8-31.2)
**Africa**	1 290 (1 280-1 300)	7.65 (6.1-9.01)	448 (356-530)	15.8 (14-17.9)	1 190 (1 160-1 230)	7.12 (5.66-8.4)	415 (328-490)	14.4 (12.8-16.4)	1 190 (1 150-1 220)	7.08 (5.63-8.35)	412 (326-487)	14.3 (12.7-16.2)
**IPD**	23.5 (18.3-29.5)	2.52 (1.65-3.48)	151 (97.7-209)	4.08 (2.74-5.65)	20.4 (15.8-25.7)	2.28 (1.48-3.17)	135 (86.6-188)	3.55 (2.36-4.94)	20.2 (15.5-25.4)	2.26 (1.47-3.14)	134 (85.8-187)	3.5 (2.34-4.88)
**nIPD Pneumonia**	88.6 (80.1-98.1)	5.13 (4.45-5.53)	297 (258-321)	4.52 (4.08-5)	81.7 (73.1-90.9)	4.84 (4.19-5.27)	280 (242-304)	4.17 (3.73-4.64)	81.2 (72.5-90.5)	4.82 (4.17-5.25)	278 (240-303)	4.13 (3.69-4.61)
**Acute otitis media**	1 180 (1 180-1 180)	0 (0-0)	0.293 (0.293-0.293)	7.22 (7.22-7.22)	1 090 (1 060-1 120)	0 (0-0)	0.272 (0.265-0.28)	6.71 (6.53-6.89)	1 090 (1 050-1 120)	0 (0-0)	0.271 (0.262-0.279)	6.65 (6.45-6.85)
**Asia**	1 210 (1 190-1 240)	5.17 (4.15-6.08)	358 (287-423)	66 (58.1-74.8)	1 180 (1 160-1 210)	5.02 (4.05-5.91)	348 (279-411)	64.7 (57.2-73.5)	1 140 (1 110-1 170)	4.91 (3.95-5.82)	340 (272-404)	61.7 (54.3-70.3)
**IPD**	36.4 (30.5-43.1)	1.76 (1.19-2.39)	125 (84-171)	18.8 (13.1-25.2)	34.9 (29.2-41.5)	1.7 (1.16-2.31)	120 (81.6-166)	18.2 (12.8-24.4)	32.9 (27.3-39.7)	1.64 (1.12-2.25)	117 (78.6-161)	17.1 (11.8-23.3)
**nIPD Pneumonia**	164 (148-182)	3.41 (2.96-3.69)	233 (202-252)	22.8 (20.6-25.3)	160 (145-178)	3.32 (2.89-3.61)	227 (197-246)	22.5 (20.4-25)	155 (139-173)	3.27 (2.83-3.56)	223 (193-243)	21.7 (19.5-24.2)
**Acute otitis media**	1 010 (1 010-1 010)	0 (0-0)	0.252 (0.252-0.252)	24.4 (24.4-24.4)	986 (977-995)	0 (0-0)	0.246 (0.243-0.248)	24 (23.8-24.1)	950 (927-973)	0 (0-0)	0.237 (0.231-0.242)	22.9 (22.4-23.5)
**Europe**	8.02 (7.96-8.09)	0.00758 (0.00549-0.00992)	0.564 (0.402-0.745)	0.395 (0.335-0.467)	7.96 (7.89-8.02)	0.00729 (0.00526-0.00953)	0.54 (0.385-0.714)	0.39 (0.331-0.459)	7.65 (7.51-7.78)	0.00712 (0.00517-0.00929)	0.527 (0.377-0.695)	0.368 (0.314-0.432)
**IPD**	0.09 (0.0717-0.111)	0.00208 (0.000952-0.00397)	0.166 (0.0724-0.315)	0.103 (0.0586-0.16)	0.0878 (0.0701-0.108)	0.00195 (0.000886-0.00376)	0.155 (0.0677-0.298)	0.0996 (0.0571-0.154)	0.0805 (0.0643-0.0994)	0.00189 (0.000859-0.00363)	0.15 (0.0653-0.286)	0.0915 (0.0523-0.142)
**nIPD Pneumonia**	0.409 (0.364-0.453)	0.0055 (0.00454-0.00595)	0.396 (0.327-0.428)	0.142 (0.127-0.157)	0.404 (0.36-0.448)	0.00534 (0.00438-0.00577)	0.383 (0.316-0.415)	0.141 (0.126-0.156)	0.384 (0.342-0.425)	0.00523 (0.00431-0.00566)	0.376 (0.31-0.406)	0.133 (0.119-0.147)

**Acute otitis media**	7.52 (7.52-7.52)	0 (0-0)	0.00188 (0.00188-0.00188)	0.15 (0.15-0.15)	7.46 (7.44-7.48)	0 (0-0)	0.00186 (0.00186-0.00187)	0.149 (0.148-0.149)	7.18 (7.06-7.3)	0 (0-0)	0.00179 (0.00176-0.00182)	0.143 (0.14-0.145)
**Latin America**	46.3 (45.5-47.2)	0.206 (0.158-0.254)	14.9 (11.3-18.5)	3.85 (2.97-4.9)	42.7 (41.5-43.9)	0.186 (0.143-0.228)	13.3 (10.2-16.5)	3.3 (2.58-4.17)	42.2 (40.9-43.5)	0.184 (0.142-0.226)	13.2 (10.1-16.4)	3.24 (2.53-4.09)
**IPD**	1.31 (1.03-1.65)	0.0723 (0.0411-0.109)	5.4 (3.03-8.18)	1.81 (1.05-2.73)	1.06 (0.83-1.34)	0.0625 (0.0357-0.0948)	4.61 (2.59-7.04)	1.45 (0.84-2.2)	1.04 (0.813-1.32)	0.0618 (0.0353-0.0937)	4.56 (2.56-6.96)	1.42 (0.821-2.15)
**nIPD Pneumonia**	5.29 (4.79-5.86)	0.134 (0.116-0.145)	9.53 (8.27-10.3)	1.23 (1.11-1.36)	4.7 (4.24-5.22)	0.123 (0.107-0.133)	8.71 (7.58-9.43)	1.1 (0.99-1.22)	4.65 (4.2-5.17)	0.123 (0.107-0.133)	8.66 (7.53-9.39)	1.08 (0.975-1.2)
**Acute otitis media**	39.7 (39.7-39.7)	0 (0-0)	0.0099 (0.0099-0.0099)	0.809 (0.809-0.809)	36.9 (35.9-37.9)	0 (0-0)	0.0092 (0.00896-0.00945)	0.759 (0.742-0.777)	36.5 (35.4-37.6)	0 (0-0)	0.0091 (0.00882-0.00938)	0.745 (0.723-0.768)
**Oceania**	9.76 (9.68-9.86)	0.0327 (0.0261-0.039)	2.14 (1.71-2.57)	0.18 (0.127-0.246)	9.29 (9.1-9.47)	0.0312 (0.0249-0.0372)	2.04 (1.62-2.45)	0.164 (0.117-0.225)	9.25 (9.06-9.44)	0.0311 (0.0249-0.0372)	2.03 (1.62-2.44)	0.163 (0.116-0.224)
**IPD**	0.132 (0.102-0.169)	0.00906 (0.00558-0.0134)	0.61 (0.371-0.911)	0.1 (0.0526-0.162)	0.12 (0.0923-0.155)	0.00845 (0.00519-0.0126)	0.567 (0.343-0.851)	0.089 (0.0464-0.144)	0.119 (0.0918-0.154)	0.00844 (0.00518-0.0125)	0.565 (0.342-0.849)	0.0885 (0.046-0.144)
**nIPD Pneumonia**	0.55 (0.497-0.609)	0.0236 (0.0206-0.0255)	1.53 (1.33-1.66)	0.0504 (0.0455-0.0557)	0.521 (0.469-0.583)	0.0227 (0.0197-0.0248)	1.47 (1.28-1.6)	0.0476 (0.0428-0.0533)	0.519 (0.467-0.581)	0.0227 (0.0197-0.0247)	1.47 (1.28-1.6)	0.0474 (0.0426-0.0531)
**Acute otitis media**	9.08 (9.08-9.08)	0 (0-0)	0.00226 (0.00226-0.00226)	0.0289 (0.0289-0.0289)	8.65 (8.49-8.81)	0 (0-0)	0.00216 (0.00212-0.0022)	0.0274 (0.0269-0.028)	8.61 (8.45-8.79)	0 (0-0)	0.00215 (0.00211-0.00219)	0.0273 (0.0268-0.0279)

*: inclusive of only 112 low- and middle-income countries

(1) **No vaccination scenario**.

(2) **PCV coverage scenario**: Using respective year's PCV coverage data from 2000 to 2019, using 2019 PCV coverage data from 2020 to 2030.

(3) **DTP coverage scenario**: Using respective year's PCV coverage data from 2000 to 2019, using 2019 DTP coverage data from 2020 to 2030.

Healthcare costs arise from hospitalisation and outpatient visits.

IPD: invasive pneumococcal diseases, which represents the sum of pneumococcal meningitis, pneumococcal non-pneumonia, non-meningitis, and invasive pneumococcal pneumonia

nIPD: non-invasive pneumococcal diseases

AOM: acute otitis media

CrI: credible interval;

DALYs: Disability-adjusted life-years;

DTP: diphtheria-tetanus-pertussis;

PCV: pneumococcal conjugate vaccine.

Table 10 presents the percentage of cases, deaths, DALYs averted compared to no vaccination scenario. PCV introduction has the potential to avert 5·1% of cases (131 (95% CrI: 89·0-172) million cases), 5·3% of deaths (697 (95% CrI: 359-1 040) thousand deaths) and 5·6% of DALYs (46·0 (95% CrI: 24·0-68·9) million DALYs) from the period 2000 to 2030.

Table 10. Percentage of cases, deaths, DALYs averted (mean, 95% CrI) by regions and time periods.

	**Percentage reduction using PCV coverage (compared to no vaccination), 2000-2030** %: $\frac{\text{(1) No vaccination} - \text{(2) PCV introduction}}{\text{(1) No vaccination}}$			**Percentage reduction using PCV coverage (compared to no vaccination), 2020-2030** %: $\frac{\text{(1) No vaccination} - \text{(2) PCV introduction}}{\text{(1) No vaccination}}$			**Percentage reduction using DPT coverage (compared to PCV coverage), 2020-2030** %: $\frac{\text{(2)PCV introduction} - \text{(3) PCV at DTP coverage}}{\text{(2) PCV coverage}}$		
**Period**	**2000-2030**			**2020-2030**			**2020-2030**		
**Region**	**Cases**	**Deaths**	**DALYs**	**Cases**	**Deaths**	**DALYs**	**Cases**	**Deaths**	**DALYs**
**Global**	5.1	5.3	5.6	9.4	14.6	14.5	5.8	4.8	5.2
**IPD**	7.9	7.2	7.5	16.6	19.9	19.6	11.7	7.3	7.9
**nIPD Pneumonia**	4.5	4.4	4.6	9.6	11.9	11.9	6.9	3.7	3.9
**Acute otitis media**	5.1	-	5.1	9.2	-	9.2	5.5	-	5.5
**Africa**	7.4	6.8	7.5	12.2	16.6	16.9	1.5	2.0	2.0
**IPD**	13.2	9.5	10.4	24.2	23.1	23.4	2.9	3.0	2.9
**nIPD Pneumonia**	7.9	5.5	6.0	14.5	13.4	13.6	1.6	1.6	1.6
**Acute otitis media**	7.2	-	7.2	11.7	-	11.7	1.5	-	1.5
**Asia**	2.6	2.9	3.0	5.7	10.1	9.9	11.0	10.0	10.3
**IPD**	4.1	3.7	3.7	10.0	13.0	12.6	17.5	14.7	15.0
**nIPD Pneumonia**	2.5	2.5	2.6	6.0	8.6	8.5	10.1	7.7	7.8
**Acute otitis media**	2.5	-	2.5	5.5	-	5.5	11.0	-	11.0
**Europe**	0.8	3.9	4.1	1.6	14.1	14.2	12.6	13.5	13.9
**IPD**	2.4	6.2	6.3	5.8	22.2	20.9	27.6	19.5	20.3
**nIPD Pneumonia**	1.1	3.0	3.2	2.7	11.0	11.3	16.6	11.5	11.4
**Acute otitis media**	0.8	-	0.8	1.5	-	1.5	12.2	-	12.2
**Latin America**	7.9	10	10.8	14.1	25.8	26.6	3.6	3.4	3.4
**IPD**	19	13.5	14.6	37.3	34.3	35.3	6.9	5.7	5.7
**nIPD Pneumonia**	11.2	8.2	8.6	22.1	21.1	21.5	3.6	2.4	2.4
**Acute otitis media**	7.0	-	7.0	12.2	-	12.2	3.5	-	3.5
**Oceania**	4.8	4.7	4.9	9.6	13.5	13.7	1.0	0.4	0.5
**IPD**	9.1	6.7	7.1	20.5	19.3	19.6	1.9	0.8	0.9
**nIPD Pneumonia**	5.2	3.9	4.0	11.8	11.2	11.3	1.1	0.3	0.3
**Acute otitis media**	4.8	-	4.8	9.3	-	9.3	1.0	-	1.0

*: inclusive of only 112 low- and middle-income countries

(1) **No vaccination scenario**.

(2) **PCV coverage scenario**: Using respective year's PCV coverage data from 2000 to 2019, using 2019 PCV coverage data from 2020 to 2030.

(3) **DPT coverage scenario**: Using respective year's PCV coverage data from 2000 to 2019, using 2019 DTP coverage data from 2020 to 2030.

IPD: invasive pneumococcal diseases, which represents the sum of pneumococcal meningitis, pneumococcal non-pneumonia, non-meningitis, and invasive pneumococcal pneumonia

nIPD: non-invasive pneumococcal diseases

AOM: acute otitis media
CrI: credible interval;
DALYs: Disability-adjusted life-years;
DTP: diphtheria-tetanus-pertussis;
PCV: pneumococcal conjugate vaccine.

REFERENCES

1. Chen C, Liceras FC, Flasche S, et al. Effect and cost-effectiveness of pneumococcal conjugate vaccination: a global modelling analysis. *The Lancet Global Health* 2019; **7**(1): e58-e67.
2. García Fariñas A, Linares-Pérez N, Clark A, et al. Cost-effectiveness of introducing a domestic pneumococcal conjugate vaccine (PCV7-TT) into the Cuban national immunization programme. *Int J Infect Dis* 2020; **97**: 182-9.
3. Pan American Health Organization (PAHO). About UNIVAC. https://www3.paho.org/provac-toolkit/tools/about-univac/ (accessed 2022-01-10 2022).
4. Wahl B, O'Brien KL, Greenbaum A, et al. Burden of *Streptococcus pneumoniae* and *Haemophilus influenzae* type b disease in children in the era of conjugate vaccines: global, regional, and national estimates for 2000–15. *The Lancet Global Health* 2018; **6**(7): e744-e57.
5. World Health Organization. WHO/UNICEF estimates of national immunization coverage.
6. World Health Organisation. Diphtheria tetanus toxoid and pertussis (DTP) vaccination coverage. 2023. https://immunizationdata.who.int/pages/coverage/dtp.html (accessed 6 April 2023).
7. Tvedskov ESF, Hovmand N, Benfield T, Tinggaard M. Pneumococcal carriage among children in low and lower-middle-income countries: A systematic review. *International Journal of Infectious Diseases* 2022; **115**: 1-7.
8. Shiri T, Datta S, Madan J, et al. Indirect effects of childhood pneumococcal conjugate vaccination on invasive pneumococcal disease: a systematic review and meta-analysis. *The Lancet Global Health* 2017; **5**(1): e51-e9.
9. van Werkhoven CH. Herd effects of child vaccination with pneumococcal conjugate vaccine against pneumococcal non-invasive community-acquired pneumonia: What is the evidence? *Human vaccines & immunotherapeutics* 2017; **13**(5): 1177-81.
10. Flasche S, Le Polain de Waroux O, O’Brien KL, Edmunds WJ. The serotype distribution among healthy carriers before vaccination is essential for predicting the impact of pneumococcal conjugate vaccine on invasive disease. *PLoS computational biology* 2015; **11**(4): e1004173.
11. Clark A, Tate J, Parashar U, et al. Mortality reduction benefits and intussusception risks of rotavirus vaccination in 135 low-income and middle-income countries: a modelling analysis of current and alternative schedules. *The Lancet Global Health* 2019; **7**(11): e1541-e52.
12. Clark A, Sanderson C. Timing of children's vaccinations in 45 low-income and middle-income countries: an analysis of survey data. *The Lancet* 2009; **373**(9674): 1543-9.
13. Ben-Shimol S, Regev-Yochay G, Givon-Lavi N, et al. Dynamics of Invasive Pneumococcal Disease in Israel in Children and Adults in the 13-Valent Pneumococcal Conjugate Vaccine (PCV13) Era: A Nationwide Prospective Surveillance. *Clinical Infectious Diseases* 2021.
14. Waight PA, Andrews NJ, Ladhani SN, Sheppard CL, Slack MP, Miller E. Effect of the 13-valent pneumococcal conjugate vaccine on invasive pneumococcal disease in England and Wales 4 years after its introduction: an observational cohort study. *The Lancet infectious diseases* 2015; **15**(5): 535-43.

15. Mackenzie GA, Hill PC, Jeffries DJ, et al. Impact of the introduction of pneumococcal conjugate vaccination on invasive pneumococcal disease and pneumonia in the Gambia: 10 years of population-based surveillance. *The Lancet Infectious Diseases* 2021; **21**(9): 1293-302.
16. Hammitt LL, Etyang AO, Morpeth SC, et al. Effect of ten-valent pneumococcal conjugate vaccine on invasive pneumococcal disease and nasopharyngeal carriage in Kenya: a longitudinal surveillance study. *The Lancet* 2019; **393**(10186): 2146-54.
17. Cutts F, Zaman S, Enwere Gy, et al. Efficacy of nine-valent pneumococcal conjugate vaccine against pneumonia and invasive pneumococcal disease in The Gambia: randomised, double-blind, placebo-controlled trial. *The Lancet* 2005; **365**(9465): 1139-46.
18. Said MA, Johnson HL, Nonyane BA, Deloria-Knoll M, O′ Brien KL, Team AAPBS. Estimating the burden of pneumococcal pneumonia among adults: a systematic review and meta-analysis of diagnostic techniques. *PloS one* 2013; **8**(4): e60273.
19. Chan J, Mungun T, Batsaixan P, et al. Direct and indirect effects of 13-valent pneumococcal conjugate vaccine on pneumococcal carriage in children hospitalised with pneumonia from formal and informal settlements in Mongolia: an observational study. *Lancet Reg Health West Pac* 2021; **15**: 100231.
20. Lucero MG, Dulalia VE, Parreño RAN, et al. Pneumococcal conjugate vaccines for preventing vaccine-type invasive pneumococcal disease and pneumonia with consolidation on x-ray in children under two years of age. *Cochrane Database of Systematic Reviews* 2004; (4).
21. Fortanier AC, Venekamp RP, Boonacker CW, et al. Pneumococcal conjugate vaccines for preventing acute otitis media in children. *Cochrane Database of Systematic Reviews* 2019; (5).
22. Gavi the Vaccine Alliance. Pneumococcal vaccine support. 2022. https://www.gavi.org/types-support/vaccine-support/pneumococcal (accessed 15 February 2022).
23. Jokinen J, Rinta-Kokko H, Siira L, et al. Impact of ten-valent pneumococcal conjugate vaccination on invasive pneumococcal disease in Finnish children--a population-based study. *PLoS One* 2015; **10**(3): e0120290.
24. Verani JR, Domingues CM, de Moraes JC, Brazilian Pneumococcal Conjugate Vaccine Effectiveness Study G. Indirect cohort analysis of 10-valent pneumococcal conjugate vaccine effectiveness against vaccine-type and vaccine-related invasive pneumococcal disease. *Vaccine* 2015; **33**(46): 6145-8.
25. Berman-Rosa M, O'Donnell S, Barker M, Quach C. Efficacy and Effectiveness of the PCV-10 and PCV-13 Vaccines Against Invasive Pneumococcal Disease. *Pediatrics* 2020; **145**(4).
26. International Vaccine Access Center (IVAC). Johns Hopkins Bloomberg School of Public Health. VIEW-hub. https://view-hub.org/map/?set=current-vaccine-intro-status&group=vaccine-introduction&category=pcv.
27. WHO. Pneumococcal conjugate vaccines in infants and children under 5 years of age: WHO position paper–February 2019. *Weekly Epidemiological Record* 2019; **94**(08): 85-103.
28. Masters NB, Wagner AL, Boulton ML. Vaccination timeliness and delay in low- and middle-income countries: a systematic review of the literature, 2007-2017. *Hum Vaccin Immunother* 2019; **15**(12): 2790-805.

29. Collaborators GL. Estimates of the global, regional, and national morbidity, mortality, and aetiologies of lower respiratory tract infections in 195 countries: a systematic analysis for the Global Burden of Disease Study 2015. *Lancet Infect Dis* 2017; **17**(11): 1133-61.
30. World Health Organization. Model IMCI handbook: Integrated management of childhood illness. WHO, 2005.
31. World Health Organisation (WHO). Pocket Book of Hospital Care for Children: Guidelines for the Management of Common Childhood Illnesses.; 2013.
32. Edmond K, Clark A, Korczak VS, Sanderson C, Griffiths UK, Rudan I. Global and regional risk of disabling sequelae from bacterial meningitis: a systematic review and meta-analysis. *The Lancet infectious diseases* 2010; **10**(5): 317-28.
33. Monasta L, Ronfani L, Marchetti F, et al. Burden of disease caused by otitis media: systematic review and global estimates. *PloS one* 2012; **7**(4): e36226.
34. Centers for Disease Control and Prevention. Pneumococcal Disease Clinical features for acute otitis media.
35. Li JZ, Winston LG, Moore DH, Bent S. Efficacy of short-course antibiotic regimens for community-acquired pneumonia: a meta-analysis. *The American journal of medicine* 2007; **120**(9): 783-90.
36. Dimopoulos G, Matthaiou DK, Karageorgopoulos DE, Grammatikos AP, Athanassa Z, Falagas ME. Short-versus Long-Course Antibacterial Therapy for Community-Acquired Pneumonia. *Drugs* 2008; **68**(13): 1841-54.
37. Tansarli GS, Mylonakis E. Systematic review and meta-analysis of the efficacy of short-course antibiotic treatments for community-acquired pneumonia in adults. *Antimicrobial agents and chemotherapy* 2018; **62**(9): e00635-18.
38. Tunkel AR, Hartman BJ, Kaplan SL, et al. Practice guidelines for the management of bacterial meningitis. *Clinical infectious diseases* 2004; **39**(9): 1267-84.
39. Bradley JS, Byington CL, Shah SS, et al. The management of community-acquired pneumonia in infants and children older than 3 months of age: clinical practice guidelines by the Pediatric Infectious Diseases Society and the Infectious Diseases Society of America. *Clinical infectious diseases* 2011; **53**(7): e25-e76.
40. Vos T, Lim SS, Abbafati C, et al. Global burden of 369 diseases and injuries in 204 countries and territories, 1990–2019: a systematic analysis for the Global Burden of Disease Study 2019. *The Lancet* 2020; **396**(10258): 1204-22.
41. United Nations. 2019 Revision of World Population Prospects. 2019.
42. Portnoy A, Jit M, Lauer J, et al. Estimating costs of care for meningitis infections in low- and middle-income countries. *Vaccine* 2015; **33 Suppl 1**: A240-7.
43. WHO. WHO-CHOICE unit cost estimates for service delivery. 2011.
44. Zhang S, Sammon PM, King I, et al. Cost of management of severe pneumonia in young children: systematic analysis. *J Glob Health* 2016; **6**(1): 010408.
45. Saokaew S, Rayanakorn A, Wu DB, Chaiyakunapruk N. Cost Effectiveness of Pneumococcal Vaccination in Children in Low- and Middle-Income Countries: A Systematic Review. *Pharmacoeconomics* 2016; **34**(12): 1211-25.
46. Giglio N, Micone P, Gentile A. The pharmacoeconomics of pneumococcal conjugate vaccines in Latin America. *Vaccine* 2011; **29 Suppl 3**: C35-42.
47. Giachetto Larraz G, Telechea Ortiz H, Speranza Mourine N, et al. [Cost-effectiveness of universal pneumococcal vaccination in Uruguay]. *Rev Panam Salud Publica* 2010; **28**(2): 92-9.

48. Haasis MA, Ceria JA, Kulpeng W, Teerawattananon Y, Alejandria M. Do Pneumococcal Conjugate Vaccines Represent Good Value for Money in a Lower-Middle Income Country? A Cost-Utility Analysis in the Philippines. *PLoS One* 2015; **10**(7): e0131156.
49. Hoshi SL, Kondo M, Okubo I. Economic evaluation of vaccination programme of 13-valent pneumococcal conjugate vaccine to the birth cohort in Japan. *Vaccine* 2013; **31**(25): 2762-71.
50. Kim SY, Lee G, Goldie SJ. Economic evaluation of pneumococcal conjugate vaccination in The Gambia. *BMC Infect Dis* 2010; **10**: 260.
51. Klok RM, Lindkvist RM, Ekelund M, Farkouh RA, Strutton DR. Cost-effectiveness of a 10- versus 13-valent pneumococcal conjugate vaccine in Denmark and Sweden. *Clin Ther* 2013; **35**(2): 119-34.
52. Komakhidze T, Hoestlandt C, Dolakidze T, et al. Cost-effectiveness of pneumococcal conjugate vaccination in Georgia. *Vaccine* 2015; **33 Suppl 1**: A219-26.
53. Maurer KA, Chen HF, Wagner AL, et al. Cost-effectiveness analysis of pneumococcal vaccination for infants in China. *Vaccine* 2016; **34**(50): 6343-9.
54. Mezones-Holguin E, Canelo-Aybar C, Clark AD, et al. Cost-effectiveness analysis of 10- and 13-valent pneumococcal conjugate vaccines in Peru. *Vaccine* 2015; **33 Suppl 1**: A154-66.
55. Newall AT, Creighton P, Philp DJ, Wood JG, MacIntyre CR. The potential cost-effectiveness of infant pneumococcal vaccines in Australia. *Vaccine* 2011; **29**(45): 8077-85.
56. Ordóñez JE, Orozco JJ. Cost-effectiveness analysis of the available pneumococcal conjugated vaccines for children under five years in Colombia. *Cost Eff Resour Alloc* 2015; **13**: 6.
57. Wu DB-C, Chaiyakunapruk N, Chong H-Y, Beutels P. Choosing between 7-, 10- and 13-valent pneumococcal conjugate vaccines in childhood: A review of economic evaluations (2006–2014). *Vaccine* 2015; **33**(14): 1633-58.
58. Mark HR, Elisabeth AMS, Albert Jan van H, et al. Cost effectiveness of pneumococcal vaccination among Dutch infants: economic analysis of the seven valent pneumococcal conjugated vaccine and forecast for the 10 valent and 13 valent vaccines. *BMJ* 2010; **340**: c2509.
59. Rubin JL, McGarry LJ, Strutton DR, et al. Public health and economic impact of the 13-valent pneumococcal conjugate vaccine (PCV13) in the United States. *Vaccine* 2010; **28**(48): 7634-43.
60. Sartori AM, de Soárez PC, Novaes HM. Cost-effectiveness of introducing the 10-valent pneumococcal conjugate vaccine into the universal immunisation of infants in Brazil. *J Epidemiol Community Health* 2012; **66**(3): 210-7.
61. Sibak M, Moussa I, El-Tantawy N, et al. Cost-effectiveness analysis of the introduction of the pneumococcal conjugate vaccine (PCV-13) in the Egyptian national immunization program, 2013. *Vaccine* 2015; **33 Suppl 1**: A182-91.
62. Sohn HS, Suh DC, Jang E, Kwon JW. Economic evaluation of childhood 7-valent pneumococcal conjugate vaccination in Korea. *J Manag Care Pharm* 2010; **16**(1): 32-45.
63. Strutton DR, Farkouh RA, Earnshaw SR, et al. Cost-effectiveness of 13-valent pneumococcal conjugate vaccine: Germany, Greece, and The Netherlands. *J Infect* 2012; **64**(1): 54-67.
64. Sundaram N, Chen C, Yoong J, et al. Cost-effectiveness of 13-valent pneumococcal conjugate vaccination in Mongolia. *Vaccine* 2017; **35**(7): 1055-63.

65. Talbird SE, Taylor TN, Knoll S, Frostad CR, García Martí S. Outcomes and costs associated with PHiD-CV, a new protein D conjugate pneumococcal vaccine, in four countries. *Vaccine* 2010; **28 Suppl 6**: G23-9.
66. Türel O, Kisa A, McIntosh ED, Bakir M. Potential cost-effectiveness of pneumococcal conjugate vaccine (PCV) in Turkey. *Value Health* 2013; **16**(5): 755-9.
67. Tyo KR, Rosen MM, Zeng W, et al. Cost-effectiveness of conjugate pneumococcal vaccination in Singapore: comparing estimates for 7-valent, 10-valent, and 13-valent vaccines. *Vaccine* 2011; **29**(38): 6686-94.
68. van de Vooren K, Duranti S, Curto A, Garattini L. Cost effectiveness of the new pneumococcal vaccines: a systematic review of European studies. *Pharmacoeconomics* 2014; **32**(1): 29-45.
69. Urueña A, Pippo T, Betelu MS, et al. Cost-effectiveness analysis of the 10- and 13-valent pneumococcal conjugate vaccines in Argentina. *Vaccine* 2011; **29**(31): 4963-72.
70. van Hoek AJ, Choi YH, Trotter C, Miller E, Jit M. The cost-effectiveness of a 13-valent pneumococcal conjugate vaccination for infants in England. *Vaccine* 2012; **30**(50): 7205-13.
71. Vučina VV, Filipović SK, Kožnjak N, et al. Cost-effectiveness of pneumococcal conjugate vaccination in Croatia. *Vaccine* 2015; **33 Suppl 1**: A209-18.
72. Aljunid S, Maimaiti N, Ahmed Z, et al. Economic Impact of Pneumococcal Protein-D Conjugate Vaccine (PHiD-CV) on the Malaysian National Immunization Programme. *Value Health Reg Issues* 2014; **3**: 146-55.
73. Blank PR, Szucs TD. Cost-effectiveness of 13-valent pneumococcal conjugate vaccine in Switzerland. *Vaccine* 2012; **30**(28): 4267-75.
74. Chuck AW, Jacobs P, Tyrrell G, Kellner JD. Pharmacoeconomic evaluation of 10- and 13-valent pneumococcal conjugate vaccines. *Vaccine* 2010; **28**(33): 5485-90.
75. Díez-Domingo J, Ridao-López M, Gutiérrez-Gimeno MV, Puig-Barberá J, Lluch-Rodrigo JA, Pastor-Villalba E. Pharmacoeconomic assessment of implementing a universal PCV-13 vaccination programme in the Valencian public health system (Spain). *Vaccine* 2011; **29**(52): 9640-8.
76. World Health Organization. Estimation of unit costs for general health services: Updated WHO-CHOICE estimates. 2011.
77. Witt WP WA, Elixhauser A. Overview of Hospital Stays for Children in the United States, 2012. 2012.
78. Duan N. Smearing Estimate: A Nonparametric Retransformation Method. *Journal of the American Statistical Association* 1983; **78**(383): 605-10.
79. Bertram MY, Lauer JA, De Joncheere K, et al. Cost-effectiveness thresholds: pros and cons. *Bull World Health Organ* 2016; **94**(12): 925-30.
80. Edejer TTT, Edejer TTT, Organization WH. Making Choices in Health: WHO Guide to Cost-effectiveness Analysis: World Health Organization; 2003.
81. Samuel C, Maria DK, Katherine LOB, et al. Global landscape of *Streptococcus pneumoniae* serotypes colonising healthy individuals worldwide before vaccine introduction; a systematic review and meta-analysis. *medRxiv* 2023: 2023.03.09.23287027.
82. Toor J, Echeverria-Londono S, Li X, et al. Lives saved with vaccination for 10 pathogens across 112 countries in a pre-COVID-19 world. *Elife* 2021; **10**: e67635.
83. Li X, Mukandavire C, Cucunubá ZM, et al. Estimating the health impact of vaccination against ten pathogens in 98 low-income and middle-income countries from 2000 to 2030: a modelling study. *The Lancet* 2021; **397**(10272): 398-408.

84. von Gottberg A, de Gouveia L, Tempia S, et al. Effects of vaccination on invasive pneumococcal disease in South Africa. *N Engl J Med* 2014; **371**(20): 1889-99.
85. Adamu AL, Ojal J, Abubakar IA, et al. The impact of introduction of the 10-valent pneumococcal conjugate vaccine on pneumococcal carriage in Nigeria. *Nature Communications* 2023; **14**(1): 2666.
86. Flasche S, Lipsitch M, Ojal J, Pinsent A. Estimating the contribution of different age strata to vaccine serotype pneumococcal transmission in the pre vaccine era: a modelling study. *BMC medicine* 2020; **18**(1): 1-12.
87. Newall AT, Jit M, Hutubessy R. Are current cost-effectiveness thresholds for low- and middle-income countries useful? Examples from the world of vaccines. *Pharmacoeconomics* 2014; **32**(6): 525-31.
